# Targeting active site residues and structural anchoring positions in terpene synthases

**DOI:** 10.3762/bjoc.17.161

**Published:** 2021-09-17

**Authors:** Anwei Hou, Jeroen S Dickschat

**Affiliations:** 1 Kekulé-Institute of Organic Chemistry and Biochemistry, University of Bonn, Gerhard-Domagk-Straße 1, 53121 Bonn, Germany

**Keywords:** biosynthesis, enzyme mechanisms, isotopes, site-directed mutagenesis, terpenes

## Abstract

The sesterterpene synthase SmTS1 from *Streptomyces mobaraensis* contains several unusual residues in positions that are otherwise highly conserved. Site-directed mutagenesis experiments for these residues are reported that showed different effects, resulting in some cases in an improved catalytic activity, but in other cases in a loss of enzyme function. For other enzyme variants a functional switch was observed, turning SmTS1 from a sesterterpene into a diterpene synthase. This article gives rational explanations for these findings that may generally allow for protein engineering of other terpene synthases to improve their catalytic efficiency or to change their functions.

## Introduction

**Figure 1:**
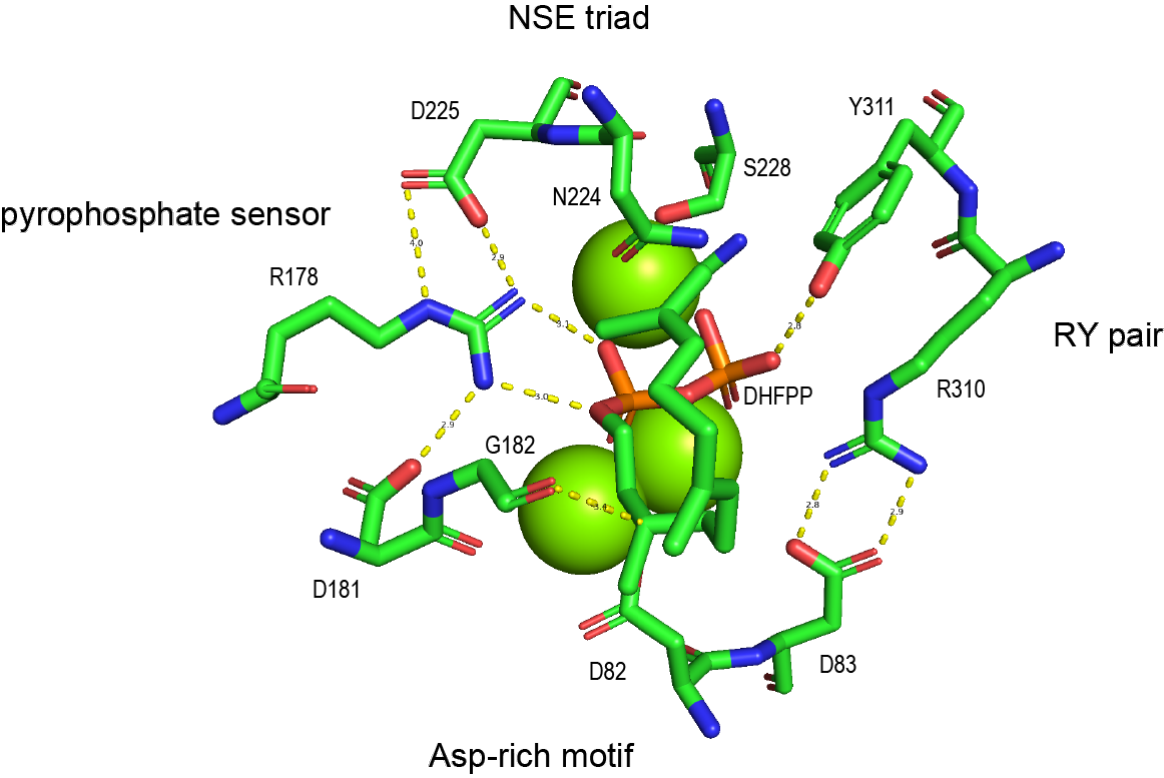
Highly conserved residues in the active site of SdS for Mg^2+^ complexation, substrate recognition and activation. DHFPP = 2,3-dihydro-FPP.

Terpenoids now span more than 90,000 known compounds, which makes them by far the largest class of natural products [1]. Despite this fact, all compounds are made from only two C_5_ building blocks, dimethylallyl diphosphate (DMAPP) and isopentenyl diphosphate (IPP), that can be fused by oligoprenyl diphosphate synthases to yield geranyl diphosphate (GPP, C_10_) as the precursor to monoterpenes, farnesyl diphosphate (FPP, C_15_) as sesquiterpene precursor, geranylgeranyl diphosphate (GGPP, C_20_) towards diterpenes, and geranylfarnesyl diphosphate (GFPP, C_25_) for sesterterpene biosynthesis. Type I terpene synthases (TPSs) activate these acyclic molecules by the abstraction of diphosphate to produce a reactive allyl cation that can initiate a cascade reaction through typical carbocation chemistry, including cyclisation reactions by intramolecular attack of an olefinic double bond to the cationic centre Wagner–Meerwein rearrangements, and proton or hydride migrations [2]. These multistep cascade reactions ultimately result in terpene hydrocarbons that are often (poly)cyclic and contain several stereogenic centres [3–4]. In some cases, water is incorporated by its nucleophilic attack at a cationic intermediate, leading to terpene alcohols [5–6] or sometimes ethers [7–8]. Substrate ionisation by TPSs is achieved through binding of the diphosphate portion to a trinuclear Mg^2+^ cluster in the active site that is itself bound to two highly conserved motifs (Supporting Information File 1, Figure S1), composed in bacterial and non-plant eukaryotic enzymes of the aspartate-rich motif DDXX(X)D around position 90 and the NSE triad ND(L,I,V)XSXX(K,R)E near position 230 (Figure 1) [9]. While the amino acid sequences of two TPSs can strongly deviate, their overall structures are very similar and constitute an α-helical fold that was first described for the FPP synthase (FPPS) from chicken (*Gallus gallus*) [10]. A helix G break first observed in tobacco 5-*epi*-aristolochene synthase with a nearby main chain carbonyl group stabilises the allyl cation of the ionised substrate [9]. The structure of bacterial selina-4(15),7(11)-diene synthase (SdS) and its comparison to other TPS structures revealed that this helix break motif is a general feature of type I TPSs [11]. Furthermore, hydrogen bondings of the substrate’s diphosphate to a highly conserved Arg residue (pyrophosphate sensor, located usually 46 residues upstream of the NSE triad, Figure 1) can be observed. Site-directed mutagenesis demonstrated that this residue is important for SdS catalysis [11]. Additional conserved residues include a Pro at the bottom of helix C causing a helix turn (21 positions upstream of the DDXX(X)D motif), a Leu (Val, Ile) at the start of helix D that with its steric bulk maintains a distance between helices D and G (14 positions upstream of the DDXX(X)D motif) [12–14], a pair of an Arg and a Glu (Asp) residue (34 positions upstream and 14 positions downstream of the pyrophosphate sensor) that form a salt bridge between helices F and G [12–14], and a conserved Asn (8 or 9 residues downstream of the NSE triad) that hydrogen bridges to the Mg^2+^ binding Glu of the NSE triad [15]. In between these structural anchors the amino acid sequences of terpene synthases seem to be quite random, only the active site is lined with mostly non-polar residues. They contour the active site and force the substrate into a certain conformation which, after substrate ionisation, determines the reaction pathway that is taken by the cationic cascade. Here we present site-directed mutagenesis experiments with the sestermobaraene synthase SmTS1 [16] that target the positions usually taken by the described structural anchors and active site contouring residues.

## Results and Discussion

### Analysis of active site residues of SmTS1

**Figure 2:**
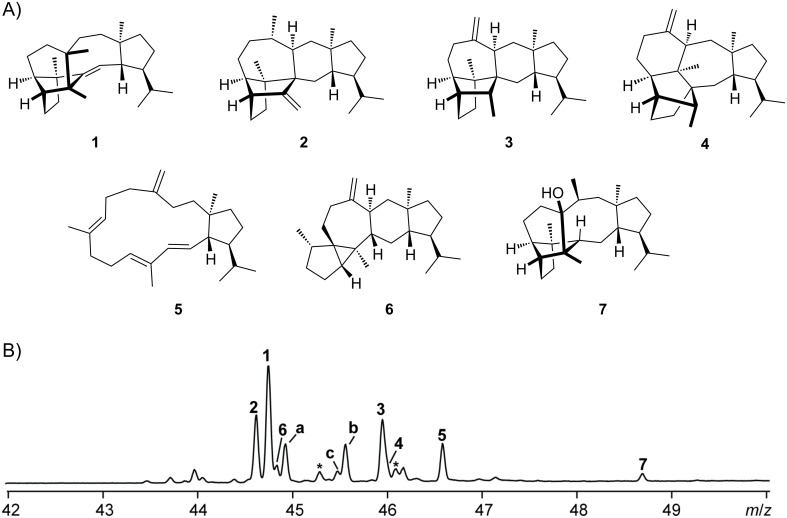
The products of SmTS1. A) Structures of sestermobaraenes A–F (**1**–**6**) and sestermobaraol (**7**). B) The total ion chromatogram of the products obtained with SmTS1 from GFPP. Peak labels **a**, **b** and **c** indicate unknown products, asterisks indicate degradation products from GFPP that are also observed without enzyme.

The recently described sestermobaraene synthase from *Streptomyces mobaraensis* (SmTS1) represents the first identified type I sesterterpene synthase (StTPS) from bacteria [16]. This enzyme converts GFPP into multiple products seven of which could be isolated and structurally characterised as sestermobaraenes A–F (**1**–**6**) and sestermobaraol (**7**) (Figure 2). SmTS1 has a low amino acid sequence identity to other characterised TPSs, with the diterpene synthase (DTS) for cattleyene from *Streptomyces cattleya* as one of the closest relatives, which shows only 29% sequence identity [17].

**Figure 3:**
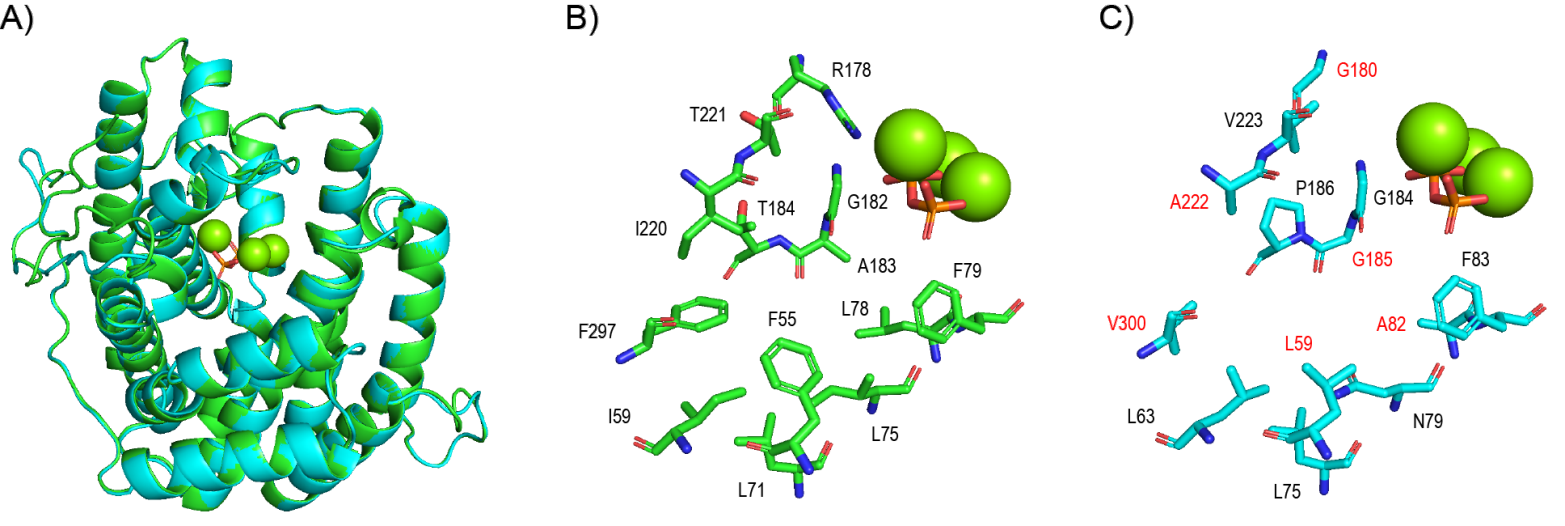
Swiss homology modelling of SmTS1. A) Superimposition of the SdS crystal structure (green) with the SmTS1 model (cyan). B) Active site residues of SdS. C) Active site residues of SmTS1. Active site residues that are smaller than in SdS are labelled in red. Green spheres represent Mg^2+^ cations and orange/red sticks show complexed diphosphate.

We have recently shown that the sum of the calculated van der Waals volumina (Σ*V*_vdW_) of the active site residues of TPSs can be easily calculated using a simple equation by Abraham and co-workers [18]. They show a clear trend, with the average values being largest for monoterpene synthases (MTPSs, Σ*V*_vdW_ = 907 ± 24 Å^3^), and then decreasing for sesquiterpene synthases (STPSs, Σ*V*_vdW_ = 855 ± 58 Å^3^) and DTPSs (Σ*V*_vdW_ = 776 ± 107 Å^3^), reaching the smallest value for StTPSs (Σ*V*_vdW_ = 733 ± 79 Å^3^) [19]. As a consequence, the available active site space will increase from MTPSs to StTPSs to fulfill the increasing space requirements to accommodate the substrate. Despite their different functions as STPS and StTPS, the crystal structure of SdS [11] can be used as a template for SmTS1 to generate a Swiss homology model [20] (template pdb code 4OKM, Figure 3A). The active site residues of SdS make up a hydrophobic cavity (Figure 3B), that is structurally reflected in the SmTS1 model (Figure 3C). Only here several amino acid residues are smaller than in the SdS active site, which explains why SmTS1 can accept the large substrate GFPP and SdS cannot. Notably, the active site residues are always located in analogous positions, as we have recently summarised in reference [19].

Besides the large active site cavity SmTS1 exhibits a few notable features within its amino acid sequence. The aspartate-rich motif, that is usually composed of DDXX(X)D and is responsible for binding of two Mg^2+^ cations (Figure 1) [11,21], is modified to N^86^DLTV in SmTS1. Similarly, the NSE triad for binding of the third Mg^2+^ [22], showing usually the sequence ND(L,I,V)XSXX(K,R)E, is changed to N^226^QRYSYFKE in SmTS1. The pyrophosphate sensor R178 [11] forms hydrogen bridges to the substrate’s diphosphate unit and to the conserved Asp in the NSE triad (Figure 1). This residue is missing in SmTS1 and instead a glycine is observed in the corresponding position (G180). Furthermore, the highly conserved Asn located eight positions downstream of the NSE triad [15] is in SmTS1 substituted by an Arg (R242). A usually conserved Trp six positions upstream of the C-terminal RY pair [23], that is itself involved in hydrogen bonds to the substrate’s diphosphate and to the second Asp of the Asp-rich motif (Figure 1), is also not observed in SmTS1, but here a Phe residue (F307) is found.

### Site-directed mutagenesis and sesterterpene synthase activity

**Figure 4:**
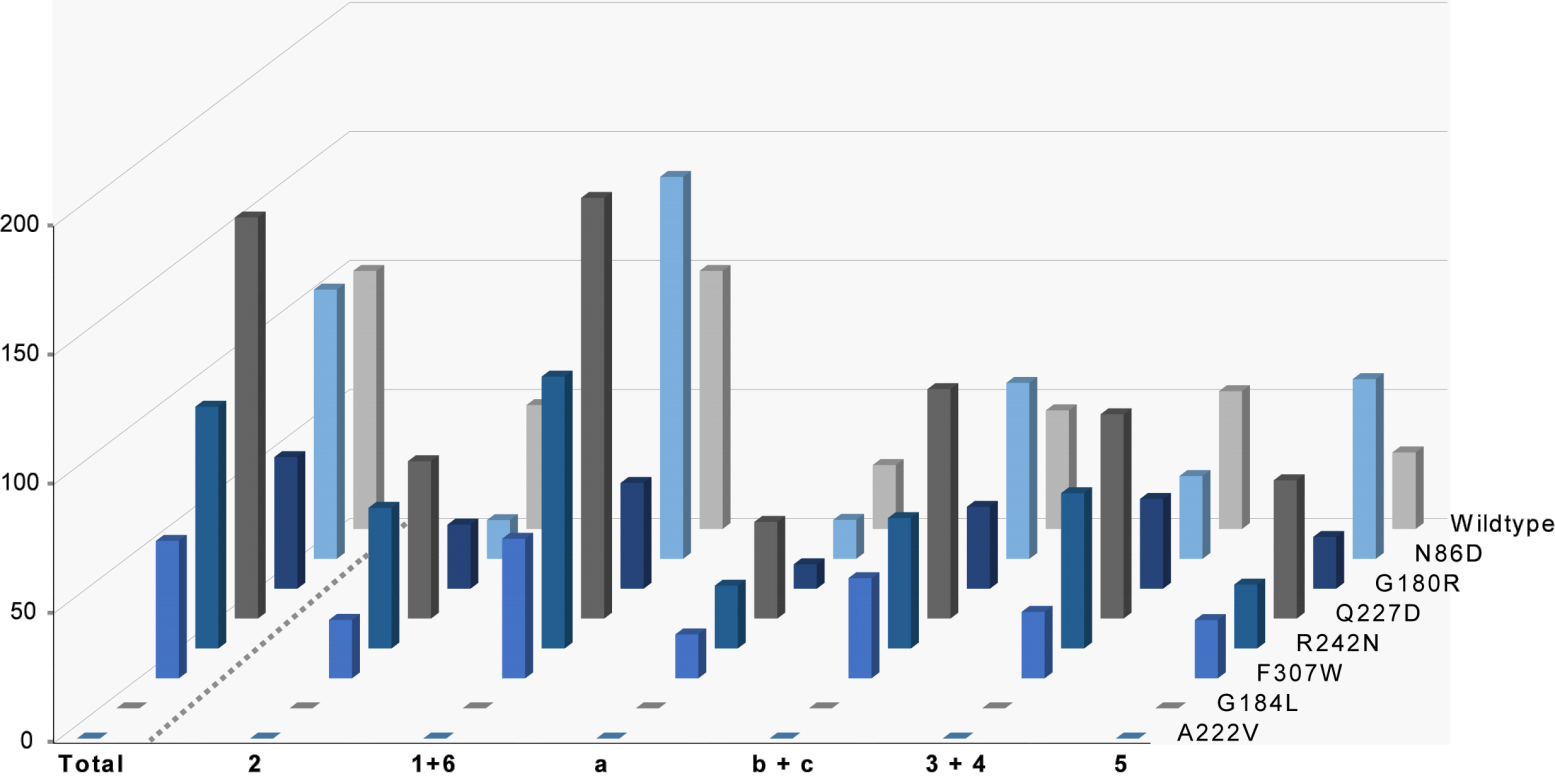
Products and relative activities of SmTS1 and its variants. Bars left of the dashed line show relative total sesterterpene production (wildtype = 100%), bars right of the dashed line show relative production of individual compounds (co-eluting **1** + **6**, **b** + **c**, and **3** + **4** are integrated together; **1** + **6** by wildtype = 100%; production of **7** was too low for accurate peak integration). Means from triplicates, for standard deviations cf. Supporting Information File 1, Table S2 and Figure S4.

To investigate possible functions of the unusual residues in SmTS1, expression constructs for the enzyme variants N86D, G180R, Q227D, R228L, R242N and F307W were made available by site-directed mutagenesis. In addition, the effect of exchanging the observed small amino acid residues lining the active site cavity in SmTS1 against larger residues was tested by construction of expression plasmids for the G184L and A222V enzyme variants. All SmTS1 derivatives were expressed, purified and adjusted to the same protein concentration (80 μg mL^−1^), with the exception of the R228L variant that was obtained with very low yields in the soluble fraction and thus not further studied (Supporting Information File 1, Figure S2). Enzyme reactions with GFPP as substrate were performed in triplicates and the relative activities were determined based on the sum of peak integrals for all sesterterpene products monitored by GC–MS (for representative chromatograms cf. Supporting Information File 1, Figure S3). The relative abundance of individual products from every variant was also analysed and compared to the wildtype (Figure 4).

The N86D enzyme variant resulted in a similar total sesterterpene production as the wildtype (104 ± 9%), showing that Asn can functionally fully substitute for the otherwise conserved Asp in this position. The relative proportions of the sesterterpenes were slightly shifted in favour of the main product **1** and compound **5**, while the production of **2** and **3** was decreased. Interestingly, the restoration of the pyrophosphate sensor in the G180R variant resulted in a decreased production (51 ± 16%), suggesting that installation of the large Arg residue blocks the available active site space for GFPP and thus disturbs the sesterterpene production by SmTS1, while the relative proportions of the individual products were similar as for the wildtype enzyme. The Q227D exchange also showed an interesting effect, causing a ca. 1.5-fold increased production (155 ± 13%), but no changes in the product proportions. The reason for this increase is not clear, but the introduced Asp in the Q227D variant is homologous to D225 of SdS that hydrogen bridges to the pyrophosphate sensor R178 (Figure 1). As SmTS1 does not contain this Arg residue, but a Gly instead, the opened space in this region could allow for direct hydrogen bonds between Q227 and the substrate’s diphosphate, which may become even stronger in the Q227D variant, explaining its higher catalytic efficiency. Future structural work on SmTS1 and its Q227D derivative is required to clarify this effect. The R242N substitution showed almost no consequences for total production (94 ± 17%) and yields of individual compounds, while for the F307W variant the overall yield dropped to 53 ± 6% with similar product ratios as for the wildtype, which may again have steric reasons. Taken together, these results demonstrate that residues found to be highly conserved and known to be critical for enzyme function in type I TPSs can in some cases be naturally substituted by other residues without consequences on the enzyme activity. Similar observations were recently made for spiroalbatene synthase from *Allokutzneria albata*, in which the otherwise highly conserved Ser within the NSE triad is naturally substituted by Gly. In this case, the G229S enzyme variant did not yield any soluble protein, possibly because the conformational flexibility of Gly is critical for correct enzyme folding [13]. At the same time our results demonstrate that such unusual residues are of interest for protein engineering and may lead to significantly increased yields, if altered to the otherwise observed conserved residues, as demonstrated for the Q227D enzyme variant.

Regarding the active site contouring residues, the G184L resulted in a completely disrupted sesterterpene biosynthesis, which supports the hypothesis that SmTS1 exhibits an unusually large active site cavity capable of taking up GFPP, while the enzyme variants with larger active site residues cannot. The G184L variant also showed no activity with any other substrate (GGPP, FPP, GPP), which underpins the previously described role of this residue for SdS as effector: Upon active site closure the main chain carbonyl group of this conserved Gly comes into close contact with C3 of the substrate (G182 in Figure 1) and assists in substrate ionisation. The introduction of steric bulk at this position blocked this movement for SdS, resulting in inactivity [11].

### Diterpene synthase activity of SmTS1 variants

**Figure 5:**
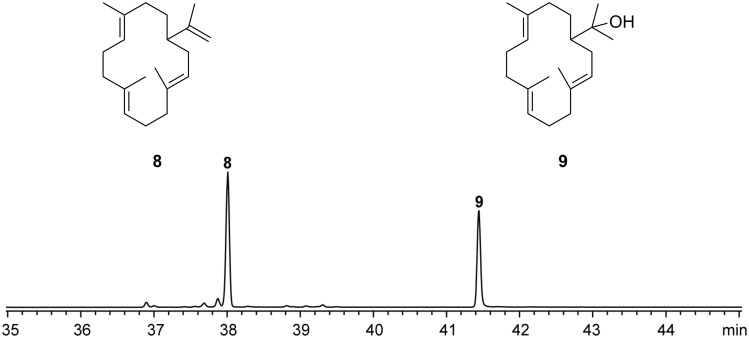
Total ion chromatogram of an extract from an incubation of GGPP with the SmTS1 A222V variant.

While the A222V enzyme variant did not convert GFPP, presumably because the active site cavity of SmTS1 becomes too narrow by this exchange for acceptance of GFPP, this exchange could still allow for the acceptance of GGPP. Incubation experiments revealed that in contrast to the wildtype, the variant A222V indeed efficiently converted GGPP into the two known diterpenes cembrene A (**8**) and nephthenol (**9**) that were identified by GC–MS (Figure 5 and Supporting Information File 1, Figure S5). While it is easy to understand that GFPP cannot enter the active site of the A222V variant, these findings raise the question why GGPP is not converted by the wildtype? A possible explanation may be that for efficient catalysis to yield a cyclic product the substrate needs to be tightly bound in the active site. If the space is too large, this may allow for too much conformational flexibility of the substrate which may prevent an efficient terpene cyclisation reaction.

**Figure 6:**
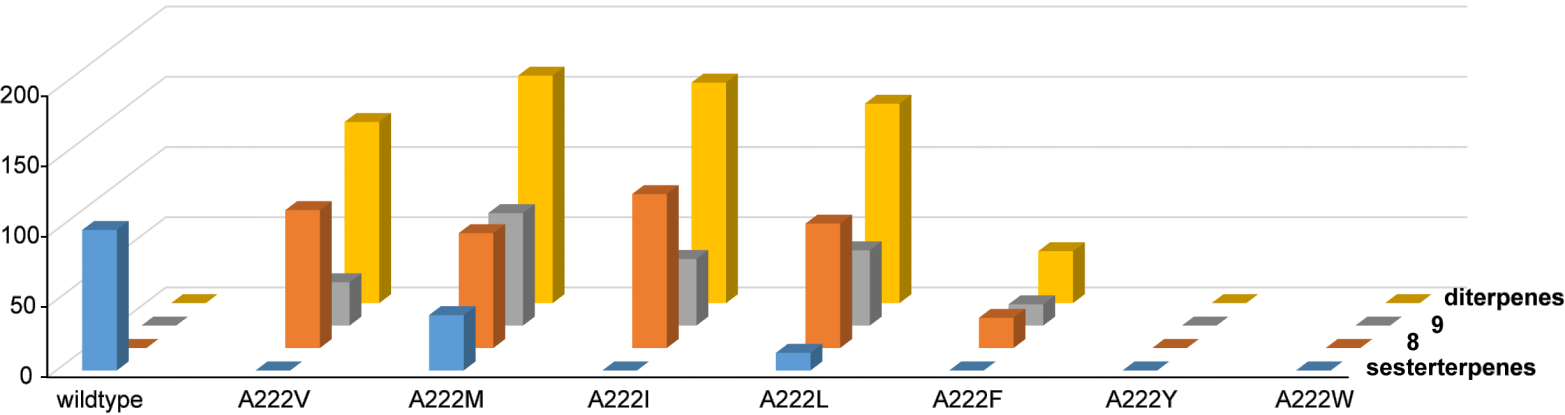
Relative activities of SmTS1 and its variants towards GFPP (blue bars) and GGPP (yellow bars), and the production of compounds **8** (red bars) and **9** (grey bars). Wildtype activity towards GFPP was set to 100%. Means from triplicates, for standard deviations cf. Table S3 and Figure S8.

The relative total production of A222V (129 ± 8%) was determined in comparison to the total production of sesterterpenes by the wildtype (= 100%, Figure 6 and Supporting Information File 1, Table S3). To further investigate the influence of bulky residues in this position, expression vectors for the SmTS1 derivatives A222M, A222I, A222L, A222F, A222Y and A222W were constructed. While the A222M (39 ± 9%) and A222L variant (13 ± 4%) retained some activity towards GFPP (Supporting Information File 1, Figure S6), all other enzyme variants for this position did not. The exchanges of A222I (157 ± 1%), A222L (142 ± 11%), and A222M (162 ± 3%) also showed a good conversion of GGPP into **8** and **9** (Supporting Information File 1, Figure S7), while A222F gave lower production (37 ± 8%). The SmTS1 derivatives A222Y and A222W were inactive with GGPP, suggesting that very large residues in this position block the space needed for acceptance of GGPP.

**Scheme 1:**
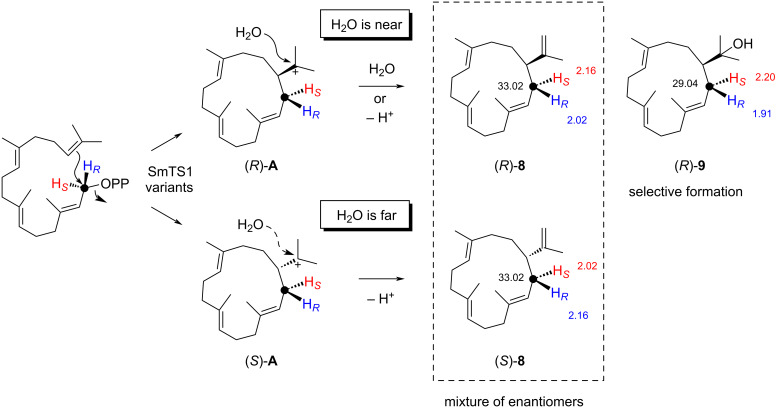
Determination of the enantiomeric composition of **8** and **9** obtained from GGPP with SmTS1 enzyme variants through enantioselective labelling with (*R*)- and (*S*)-(1-^13^C,1-^2^H)GGPP. Compound **8** is obtained with different enantiomeric ratios through (*R*)- and (*S*)-**A**. Compound **9** may be obtained with a high enantioselectivity, because an active site water could be near to the cationic centre in (*R*)-**A**, but distant in (*S*)-**A**.

To determine the absolute configurations of **8** and **9**, large scale enzyme reactions with GGPP were performed. Compound **8** was isolated from the A222V variant and **9** from the A222M variant and their structures were ultimately confirmed by NMR spectroscopy (Supporting Information File 1, Tables S4 and S5, Figures S9–S24). The optical rotation of **9** ([α]_D_^25^ = −25.1 (*c* 0.43, CH_2_Cl_2_)) pointed to the structure of (*R*)-nepthenol (lit.: [α]_D_^20^ = −31 (*c* 0.61, CHCl_3_) [24]), while the specific rotation of isolated **8** ([α]_D_^20^ = +1.5 (*c* 0.55, CH_2_Cl_2_); lit. for (*S*)-**8**: [α]_D_^20^ = +12 (*c* 0.1, CHCl_3_) [13]) revealed that this product was nearly a racemate. This unexpected finding is explainable by two different cyclisation modes of GGPP to the (*R*)- and the (*S*)-cembranyl cation (**A**, Scheme 1). The cationic centre of (*R*)-**A** may be in close proximity to an active site water, which may be able to attack at (*R*)-**A** to form (*R*)-**9**, while the distance to the cation in (*S*)-**A** is too large, preventing its attack to form (*S*)-**9**. In contrast, the formation of **8** only requires deprotonation that seems to be possible for both intermediates (*R*)- and (*S*)-**A**, explaining why compound **8** is nearly racemic.

**Figure 7:**
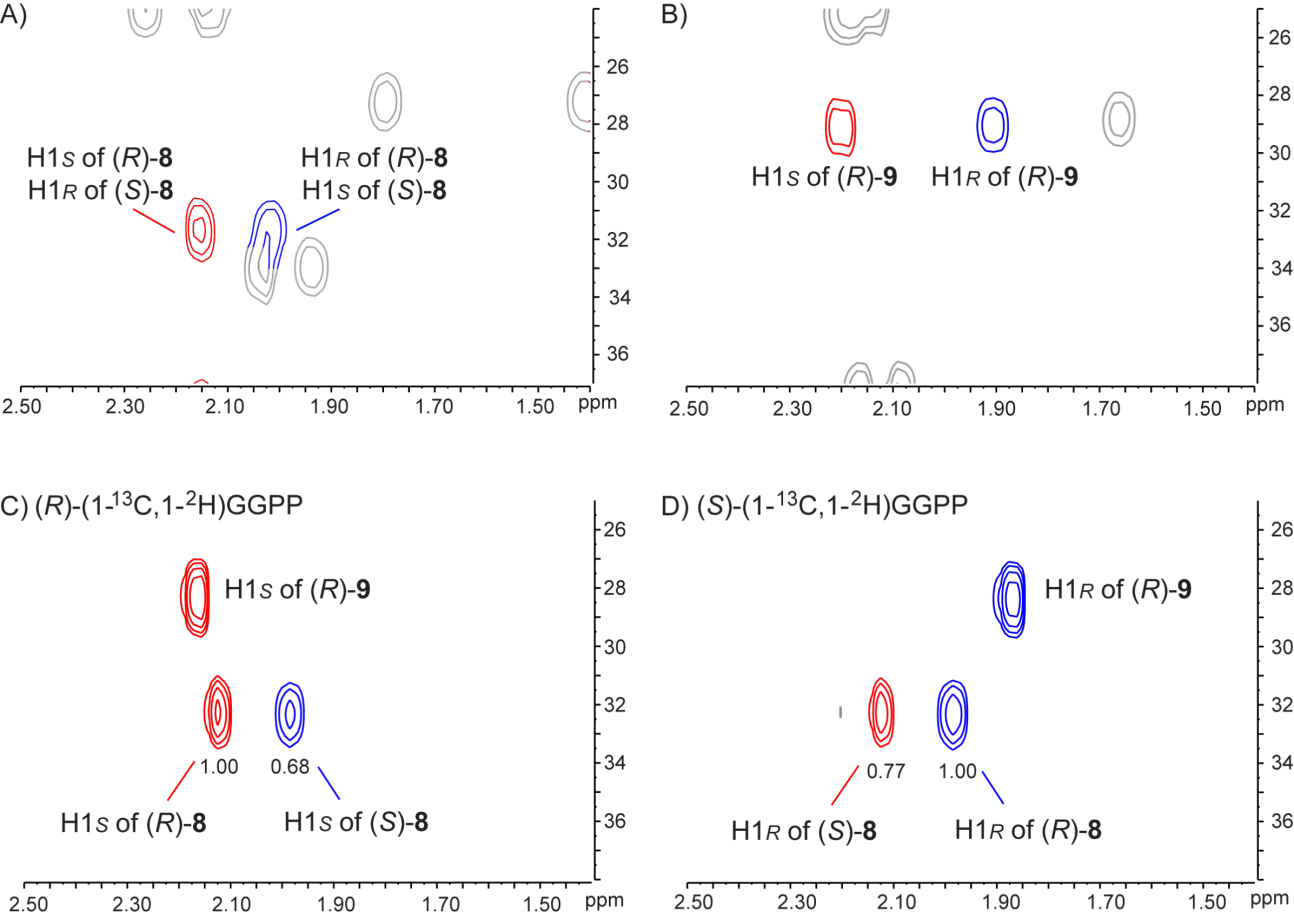
Determination of the absolute configuration of compounds **8** and **9**. Partial HSQC spectra of A) unlabelled **8**, B) unlabelled **9**, C) the mixture of labelled **8** and **9** obtained from (*R*)-(1-^13^C,1-^2^H)GGPP, and D) the mixture of labelled **8** and **9** obtained from (*S*)-(1-^13^C,1-^2^H)GGPP. The colour of cross peaks in blue and in red refers to the hydrogens in Scheme 1 of same colour.

As the gaschromatographic analysis using a chiral stationary phase did not show any resolution for the enantiomers of **8** or **9**, their enantiomeric composition was determined using the enantioselectively deuterated substrates (*R*)- and (*S*)-(1-^13^C,1-^2^H)GGPP [25]. Their conversion into different enantiomers of **8** and **9** will lead to incorporation of deuterium into diastereotopic hydrogen positions that can be distinguished by NMR spectroscopy. Herein, the additional ^13^C-labellings allow for a sensitive analysis by HSQC spectroscopy (Figure 7). Conversion of both substrates (*R*)- and (*S*)-(1-^13^C,1-^2^H)GGPP with the SmTS1 A222M variant showed the incorporation of deuterium into only one of the diastereotopic hydrogens, indicating that (*R*)-**9** is formed in an enantiomerically pure form, while for **8** from (*R*)-(1-^13^C,1-^2^H)GGPP an enantiomeric ratio of 1.00:0.68 (19% ee) and from (*S*)-(1-^13^C,1-^2^H)GGPP an enantiomeric ratio of 0.77:1.00 (13% ee) was observed, pointing to the formation of (*R*)-**8** with 16% ee (average of both experiments).

The enantiomeric composition of the products **8** and **9** from the other enzyme variants was analysed following the same strategy, revealing that (*R*)-nephthenol was produced with high enantioselectivity also by the SmTS1 derivatives with A222V, A222L, A222I and A222F exchange (Supporting Information File 1, Figure S25). For cembrene A the enantiomeric composition was found to be different for each enzyme variant, yielding (*R*)-**8** from the A222F variant with high selectivity (94% ee), but mainly (*S*)-**8** from A222V (37% ee), A222L (7% ee) and A222I (32% ee).

## Conclusion

Terpene synthases contain several well-known highly conserved motifs and single residues that are believed to be generally important for structure and function. As we show here, in special cases such as the sestermobaraene synthase SmTS1 from *Streptomyces mobaraensis* it is possible that some of the usually conserved residues are naturally exchanged, but the enzyme retains its activity. Site-directed mutagenesis with installation of the otherwise conserved residue can lead to an improved activity, as shown for the Q227D variant. In other cases, a loss of activity is observed, e.g., in SmTS1 a re-installation of the missing pyrophosphate sensor in the G180R variant reduced activity towards GFPP, likely because of the steric bulk introduced by this exchange. We have also demonstrated that exchanges within the non-polar residues lining the active site can lead to a functional switch. For SmTS1 these residues are comparably small, and their exchange by larger residues can lead to a loss of activity with GFPP, as demonstrated for the A222V variant. It is interesting to note that this exchange at the same time leads to DTPS activity, while for the wildtype no GGPP conversion is observed. These findings will assist in future protein engineering for improved activity and functional switches in other microbial terpene synthases.

## Supporting Information

File 1 Amino acid sequence alignment, details about the mutagenesis, purification and analytical data.
