# Supplementary material for: Targeting active site residues and structural anchoring positions in terpene synthases
Source: Beilstein J Org Chem. 2021 Sep 17;17:2441–9. doi: 10.3762/bjoc.17.161 (PMC8450962; doi:10.3762/bjoc.17.161)
Supplement: File 1 — Amino acid sequence alignment, details about the mutagenesis, purification and analytical data. [file Beilstein_J_Org_Chem-17-2441-s001.pdf]

BEILSTEIN JOURNAL OF ORGANIC CHEMISTRY

Supporting Information

for

Targeting active site residues and structural anchoring positions in terpene synthases

Anwei Hou and Jeroen S. Dickschat

Beilstein J. Org. Chem. **2021,** *17,* 2441–2449. doi:10.3762/bjoc.17.161

Amino acid sequence alignment, details about the mutagenesis, purification and analytical data

License and Terms: This is a supporting information file under the terms of the Creative Commons Attribution License (https://creativecommons.org/licenses/by/4.0). Please note that the reuse, redistribution and reproduction in particular requires that the author(s) and source are credited and that individual graphics may be subject to special legal provisions.
The license is subject to the *Beilstein Journal of Organic Chemistry* terms and conditions: (https://www.beilstein-journals.org/bjoc/terms)

```
Consensus                                    ----------------------MXMPQDPDFXLPFPSXXHPXLAXARXXAXAWVRXXGLV   39
Selina-4(15),7(11)-diene (S. pristinaespiralis) ---------------------MEPELTVPPLFSPIRQAIHPKHADIDVQTAAWAETFRI-   39
Polytrichastrene A (C. polytrichastri)       -----------MKTSISNEEFYAGLQQLPKPKYPFPDSMHPDFQQQREEYYDWIDREYIF   50
Spiroviolene (S. violens)                    --------------------MTVNEIDLPPIFCPLESARHPRAHLVDERAREWIRTSPMC   41
a-Amorphene (S. viridochromogenes)           --MSTTHEEIALAGPDGIPAVDLRDLIDAQLYMPFPFERNPHASEAAAGVDHWLSTWGLT   59
Germacrene A (M. marina)                     ---------------------MRDFALSALHEPPFPSRCHPAVDLAAVQSDAWVRAFGLA   40
Caryolan-1-ol (S. griseus subsp. griseus)    ---------------------MSQITLPAFHMPFQSAGCHPGLAETREAAWEWAAAEGLD   40
epi-Cubenol (S. griseus subsp. griseus)      ------------------------------------------MAHTRGHLDSWTRRTGLV   19
7-epi-a-Eudesmol (S. viridochromogenes)      ------------------------MPQDVRFDLPFETPVSKHLESARARHLRWVWEMRLV   37
Avermitilol (S. avermitilis)                 ------------------------MPQDIDFGLPAPAGISPGLEATRRHNLGWVRRLGLV   37
Pentalenene (S. exfoliatus)                  ------------------------MPQDVDFHIPLPGRQSPDHARAEAEQLAWPRSLGLI   37

Consensus                                    XSXEAREXXAXXDXADLAARXYPHAXX-AXLDLLADWIAWLFLFDDQFDD-GPLGRRPXR   96
Selina-4(15),7(11)-diene (S. pristinaespiralis) GSEELRGKLVTQDIGTFSARILPEGRE-EVVSLLADFILWLFGVDDGHCEEGELGHRPGD   97
Polytrichastrene A (C. polytrichastri)       HSKEAREKHKKHNLTDIASRGCPSLKTFAELRPLASYAANGAMMDDYWDTCTHSE-----  104
Spiroviolene (S. violens)                    TTDEERTWVAASCSTDFFARFAPDAATDDRLLWTSLWVYWGFAFDDHRCDNGPFSNRPAA  100
a-Amorphene (S. viridochromogenes)           DDPAVAAMISCTRPAELAAFNGPDMDS-GLLQIAANQIAYQFVFDDRAEDIGRHSPGR--  115
Germacrene A (M. marina)                     GDQAGHRRLAGARAAELAARACPEASP-SGLRLLTDLINWLFVVDDACDDDGLGAAPTL-   97
Caryolan-1-ol (S. griseus subsp. griseus)    LSVPARRKMIRTRPELWISLIFPQATQ-AHLDLFCQWLFWAFLVDDEFDDGPAGRDPLM-   97
epi-Cubenol (S. griseus subsp. griseus)      HRESARNRFEQADFGAFVGMVYPTADE-EHLDLVADWFVWLFLVDDQLDDGHLGRSPDR-   76
7-epi-a-Eudesmol (S. viridochromogenes)      HSREGFEEYRSWDLPQAAARTYPHASA-DDMVVLMNWFSLAFLFDDQFDASRPDRADR--   93
Avermitilol (S. avermitilis)                 GDGPSLAWYTSWDMPRLAACGFPHARG-AALDLCADAMAFFFVFDDQFDGPLGRDPAR--   93
Pentalenene (S. exfoliatus)                  RSDAAAERHLRGGYADLASRFYPHATG-ADLDLGVDLMSWFFLFDDLFDGPRGENPED--   93

Consensus                                    LAXXLXRLXAVLDGP-G-P----XXXAXPXAAALADLWRRTXXGMTPAWVARFAXXLREY  150
Selina-4(15),7(11)-diene (S. pristinaespiralis) LAGLLHRLIRVAQNP-EAP----MMQDDPLAAGLRDLRMRVDRFGTAGQTARWVDALREY  152
Polytrichastrene A (C. polytrichastri)       MMEISKRIMLLLTGE-DTN---EPTDNGIFHQFWVLRQDCLTCEMPERLYKKFIKSLNEV  160
Spiroviolene (S. violens)                    FSALAGRVQRALEAP-SAR-----DESDGFIPALQEIAAQFRSFGTPLQVRRFAAAHRAW  154
a-Amorphene (S. viridochromogenes)           LLPMLSESVAILRDG-QPP-------TTPLGAALADLHRQVQERCTPAQAARWAWNSREY  167
Germacrene A (M. marina)                     LAPTLAGLLAVLDRH-GDPEVPVPPDGGPVAVALHDLIRRARDRNRPAHLLRLVSQLREY  156
Caryolan-1-ol (S. griseus subsp. griseus)    CERAIARLVDVFDGA-A-P-------NGPMERALAGLRDRTCRGRSPQWNRQFRRDTAAW  148
epi-Cubenol (S. griseus subsp. griseus)      VRDVVDRMRAVVDGS-A-PEVLPDEDAPAAVTALVDLWKRTMPEAAPHWRTRFAWHLVTY  134
7-epi-a-Eudesmol (S. viridochromogenes)      IAEVARELIVTPLRPAGTP----PRVACPITLAWTEVWKHLSHGMSLTWQSRFAASWGRF  149
Avermitilol (S. avermitilis)                 AARVCRRLTGIVHGA-G-P----GPGADACSAAFADVWARSTDGAHPGWVARTAHEWEYY  147
Pentalenene (S. exfoliatus)                  TKQLTDQVAAALDGP-L-P----DT-APPIAHGFADIWRRTCEGMTPAWCARSARHWRNY  146
```

```
Consensus                                      L-XXYXWEAVNRAXGTVP--XLAEYLAMRRHTXGVQPFLDLXERAAGI-EXPAXXXXHPA  206
Selina-4(15),7(11)-diene (S. pristinaespiralis)  F-FSVVWEAAHRRAGTVP--DLNDYTLMRLYDGATSVVLPMLEMGHGY-ELQPYERDRTA  208
Polytrichastrene A (C. polytrichastri)         L-IGYADERVYYRTNTIP--PLAVYLLIREATSGAQPFCKYVAMQKEYRQLPDDVLEHSH  217
Spiroviolene (S. violens)                      L-SGVTWQIGNAAAGRMP--GLDEYVAMRLLSAGGEPPFAMLELATGL-EVPAQDLERPA  210
a-Amorphene (S. viridochromogenes)             V-HGLLYEAVAQAHPAPV--ESGLCRSIRSLIAGVEPFYPLCEAAQRC-ELAPEELHHPA  223
Germacrene A (M. marina)                       L-LALLWEAANRERRRVP--GVAEYVQMRRHTGGVRPSFTVTDLARPS-APRAEQRVASA  212
Caryolan-1-ol (S. griseus subsp. griseus)      L-WTYYAEAVERAAGQVP--SRAEFAKHRRDSVAMQPFLCLHEITAGI-DLPDSARSLPA  204
epi-Cubenol (S. griseus subsp. griseus)        LTTATTWEAGNRAEDVVP--SEETYIAKRRHTGAIHVCMDLIEIVAGI-DAPESVHNDPR  191
7-epi-a-Eudesmol (S. viridochromogenes)        L-EAHCEEVDLAARGLEGTLGLVEFTEFRRRTVGIHHSIDAGERSRGF-EVPAQAMAHPV  207
Avermitilol (S. avermitilis)                   F-AAQAHEAINRLRGTPG--DMESYLQVRRGIAGTDLPLSLGERAAGI-TVPAAAFHSPQ  203
Pentalenene (S. exfoliatus)                    F-DGYVDEAESRFWNAPCD-SAAQYLAMRRHTIGVQPTVDLAERAGRF-EVPHRVFDSAV  203

Consensus                                      MRALRELAADVIGWCNDIFSLEKEX-RRGXMHNLVXVLRRERGXSLQEAVDAAARXXXRR  265
Selina-4(15),7(11)-diene (S. pristinaespiralis)  VRAVAEMASFIITWDNDIFSYHKERRGSGYYLNALRVLEQERGLTPAQALDAAISQRDRV  268
Polytrichastrene A (C. polytrichastri)         IQRLHTLCAMMIGIHNDIISLPKELHREGDTMNLVKVLQQEHKTSINEAYMMALELHDNY  277
Spiroviolene (S. violens)                      VRALTEMAIMVAALDNDRHSLRKELARGQTDQNVYSVLMQETGLPLQEAVAAATRLRDRV  270
a-Amorphene (S. viridochromogenes)             MRRLSRLSADAAVWIPDLFSAVKEQ-RAGGMINLALAYRRTHRCSLPAAVTLAVRHINST  282
Germacrene A (M. marina)                       LTVLDALATDLVCWCNDLFSYGKERGTVPEAHNLITTIAGETGQDESAALHAAANRFNKA  272
Caryolan-1-ol (S. griseus subsp. griseus)      YIALRNAVTDHSGLCNDICSFEKEA-ALGYEHNAVRLIQRDRGSTLQEAVDEAGIQLARI  263
epi-Cubenol (S. griseus subsp. griseus)        FITALEAACNHVCWANDVYSFEKEQ-VLGEIHNLVHLVRHHRGLGEQQALDHVAERLAME  250
7-epi-a-Eudesmol (S. viridochromogenes)        MERMRDLAADTIGFMNDIHSFEREK-RRGDGHNLIAVLRRERGCSWQEATDEAYRMTIAR  266
Avermitilol (S. avermitilis)                   LRIMREAAIDVTLMCNDVYSLEKEE-ARGDMDNLVLVIEHARRCTRDEAVTAARGEVARR  262
Pentalenene (S. exfoliatus)                    MSAMLQIAVDVNLLLNDIASLEKEE-ARGEQNNMVMILRREHGWSKSRSVSHMQNEVRAR  262

Consensus                                      LERFLXLEEEL-PELXPEL---GXX-----------LDGXRGWIRGNYDWXR-XXXRYXA  309
Selina-4(15),7(11)-diene (S. pristinaespiralis)  MCLFTTVSEQLAEQGSPQLR--QY------------LHSLRCFIRGAQDWGI-SSVRYTT  313
Polytrichastrene A (C. polytrichastri)         LKEFLLLQENL-PSFD------KWQNMVYDY-----VQDLGIMVAGVYAWHTNDTTRYVN  325
Spiroviolene (S. violens)                      LLRFMAVHDRVRPGAGLELS--TY------------LQGLRYGIRGNAEWGL-RVPRYLS  315
a-Amorphene (S. viridochromogenes)             IREFEDLYGEVRPELSPSGI--GY------------VEGMAGWIRGCYFWSR-TVPRYAD  327
Germacrene A (M. marina)                       LATYAERDAAL-SAVADE----GMRAF---------LDTRRDWIRATYDWSR-AAGRYA-  317
Caryolan-1-ol (S. griseus subsp. griseus)      AERVQRAERELIEEIEAA----GIDGPTRTALERC-VRDYRGLVRGDFDYHA-RAERYTR  317
epi-Cubenol (S. griseus subsp. griseus)        TERFLTAEDEL-LELYPELS--GMLVPY--------LDGMRSWMRGNLDWSR-QTPRYNP  298
7-epi-a-Eudesmol (S. viridochromogenes)        LDEYLELQERV-PQMCDEL---RLDEAQRDGVRLG-VEAIQHWINGNYEWAL-TSGRYAA  320
Avermitilol (S. avermitilis)                   VIRFEQLAREV-PALCAQL---GLSAVERAHVDTY-LGVMEAWMSGYHAWQT-QTRRYTG  316
Pentalenene (S. exfoliatus)                    LEQYLLLESCL-PKVGEIY---QLDTAEREALERYRTDAVRTVIRGSYDWHR-SSGRYDA  317
```

```
Consensus                                   ADXVAAXX--------------XRGEXDXLXXVAWWWDXAXXXXRSVRRQVPAQRSA     352
Selina-4(15),7(11)-diene (S. pristinaespiralis)   PDDPANMPSVFTDVPTD-----DSTEPLDIPAVSWWWDLLAEDARSVRRQVPAQRSA     365
Polytrichastrene A (C. polytrichastri)      GGYVDAEFI-------------TQGE-------------------------------     338
Spiroviolene (S. violens)                   LGRVPDPMDEAPLEWAESPADDDRSAPRGLPTVAWWWDDALLGV-------------     359
a-Amorphene (S. viridochromogenes)          TLTAPAGL-------------------------------------------------     335
Germacrene A (M. marina)                    ---------------------------------------------------------     316
Caryolan-1-ol (S. griseus subsp. griseus)   PDLVELD---------------ERDSLSRHFAA------------------------     335
epi-Cubenol (S. griseus subsp. griseus)     ADVGQYEEPEEYLEETVLGVPPARSETAAPAPCGAEAPRAR----------------     339
7-epi-a-Eudesmol (S. viridochromogenes)     AKEGAVATAELA----------GRGSVDDLLTV------------------------     343
Avermitilol (S. avermitilis)                APHVLPST--------------GPGYFDEVLPT------------------------     335
Pentalenene (S. exfoliatus)                 EFALAAG---------------AQGYLEELGSSAH----------------------     337
```

Figure S1. Amino acid sequence alignment some characterised sesqui- and diterpene synthases. Highly conserved residues and motifs are marked in yellow.

Site-directed mutagenesis

The site mutations were performed by the overlap extension PCR (OE-PCR) method.[1] The template was the wildtype gene cloned into the pYE-Express expression vector.[2] The mutational primers are listed in Table S1, the polymerase was purchased from NEB (Q5® High-Fidelity DNA polymerase, Ipswich, Massachusetts, USA). The first-round PCRs for individual amplification of the left and the right part of the mutated gene followed the program: 1) 98 °C for 30 s; 2) 98 °C for 10 s, 67 °C for 30 s, 72 °C for 40 s; repeated 32 times; 3) 72 °C for 2 min. The PCR products were analyzed by gel electrophoresis and purified by the Wizard® SV Gel and PCR Clean-Up System (Promega, Madison, Wisconsin, USA). The obtained overlapping fragments were then mixed and were used for the next round PCRs over two steps. Step 1: 1) 98 °C for 30 s; 2) 98 °C for 10 s, 64 °C for 30 s, 72 °C for 45 s; repeated 5 times; 3) 72 °C for 2 min. Step 2: addition of primers 28B8 and 28B9 for amplification of the whole *SmTS1* gene carrying additional homology arms for cloning into the pYE-Express expression vector by homologous recombination in yeast. The PCR was then continued: 1) 98 °C for 30 s; 2) 98 °C for 10 s, 67 °C for 30 s, 72 °C for 45 s; repeated 34 times; 3) 72 °C for 2 min. The mutated genes were then purified by the Wizard® SV Gel and PCR Clean-Up System.

The mutated genes were cloned into the pYE-Express expression vector by homologous recombination in yeast following the standard PEG/LiOAc/salmon sperm protocol.[3,4] *S. cerevisiae* cultures were grown on SM-URA plates for 3 days. Single colonies were then collected, and the plasmid DNA was isolated by using the Zymoprep™ Yeast Plasmid Miniprep II kit (Zymo Research, Irvine, CA, USA). The isolated plasmids were transformed into *E. coli* BL21(DE3) electrocompetent cells by electroporation. Cells were plated on LB medium (kanamycin) and grown at 37 °C over night. Single colonies were picked and used to inoculate 3 mL LB cultures (kanamycin). After incubation at 37 °C for 12 h, the cells were collected by centrifugation (5000 rpm × 5 min), and the plasmids were isolated by the PureYield™ Plasmid Miniprep System (Promega) and checked by sequencing. The transformants containing the correct mutations were stored in 20% glycerol at −78 °C and used for protein expression.

Table S1. Primers used for site-directed mutagenesis of SmTS1.

enzyme variant	primer	nucleotide sequence (5‘ → 3‘)[a]
SmTS1 wildtype	28B8	**GGCAGCCATATGGCTAGCATGACTGGTGGA**GTGACGCTCAACCCGGTC
	28B9	**TCTCAGTGGTGGTGGTGGTGGTGCTCGAGT**CTATCCCAGGTCTCGTTGTCG
N86D		
fragment 1	28B6	GTGACGCTCAACCCGGTC
	32H8	CGGGGACGGTGAGGTCGTCGGCGAGGAAGGCCCAG
fragment 2	32H7	CTGGGCCTTCCTCGCCGACGACCTCACCGTCCCCG
	28B7	CTATCCCAGGTCTCGTTGTCG
G180R		
fragment 1	28B6	GTGACGCTCAACCCGGTC
	32I1	GGCCTCCGACGGCGTAACGGAGGGTGAGGTGTTCGTTGAC
fragment 2	32H9	GTCAACGAACACCTCACCCTCCGTTACGCCGTCGGAGGCC
	28B7	CTATCCCAGGTCTCGTTGTCG
Q227D		
fragment 1	28B6	GTGACGCTCAACCCGGTC
	32I3	CGGATTCTTTGAAGTAGGAGTACCGATCGTTGTCGAATACCGCGGTGG
fragment 2	32I2	CCACCGCGGTATTCGACAACGATCGGTACTCCTACTTCAAAGAATCCG
	28B7	CTATCCCAGGTCTCGTTGTCG
R228L		
fragment 1	28B6	GTGACGCTCAACCCGGTC
	32I5	GTGCGGATTCTTTGAAGTAGGAGTACAGCTGGTTGTCGAATACCGCG
fragment 2	32I4	CGCGGTATTCGACAACCAGCTGTACTCCTACTTCAAAGAATCCGCAC
	28B7	CTATCCCAGGTCTCGTTGTCG
R242N		
fragment 1	28B6	GTGACGCTCAACCCGGTC
	32I7	GAGGATGGTGTCGAACATGCTGTTTTTCGGTTGAGCGTGTGCG
fragment 2	32I6	CGCACACGCTCAACCGAAAAACAGCATGTTCGACACCATCCTC
	28B7	CTATCCCAGGTCTCGTTGTCG
F307W		
fragment 1	28B6	GTGACGCTCAACCCGGTC
	32I9	GAGGGCCTTGGCCGCCCAGGTGAGATGGCCGCTGAG
fragment 2	32I8	CTCAGCGGCCATCTCACCTGGGCGGCCAAGGCCCTC
	28B7	CTATCCCAGGTCTCGTTGTCG
G184L		
fragment 1	28B6	GTGACGCTCAACCCGGTC
	33A4	GGTCGCTTCCGGGCCTAAGACGGCGTAACCGAGGG
fragment 2	33A3	CCCTCGGTTACGCCGTCTTAGGCCCGGAAGCGACC
	28B7	CTATCCCAGGTCTCGTTGTCG
A222V		
fragment 1	28B6	GTGACGCTCAACCCGGTC
	33A2	GTACCGCTGGTTGTCGAATACCACGGTGGTCATGGCGG
fragment 2	33A1	CCGCCATGACCACCGTGGTATTCGACAACCAGCGGTAC

	28B7	CTATCCCAGGTCTCGTTGTCG
A222M		
fragment 1	28B6	GTGACGCTCAACCCGGTC
	34D4	GTACCGCTGGTTGTCGAATACCATGGTGGTCATGGCGGC
fragment 2	34D3	GCCGCCATGACCACCATGGTATTCGACAACCAGCGGTAC
	28B7	CTATCCCAGGTCTCGTTGTCG
A222L		
fragment 1	28B6	GTGACGCTCAACCCGGTC
	34D2	GTACCGCTGGTTGTCGAATACCAGGGTGGTCATGGCGGC
fragment 2	34D1	GCCGCCATGACCACCCTGGTATTCGACAACCAGCGGTAC
	28B7	CTATCCCAGGTCTCGTTGTCG
A222I		
fragment 1	28B6	GTGACGCTCAACCCGGTC
	34C9	GTACCGCTGGTTGTCGAATACAATGGTGGTCATGGCGGC
fragment 2	34C8	GCCGCCATGACCACCATTGTATTCGACAACCAGCGGTAC
	28B7	CTATCCCAGGTCTCGTTGTCG
A222F		
fragment 1	28B6	GTGACGCTCAACCCGGTC
	34C7	GTACCGCTGGTTGTCGAATACAAAGGTGGTCATGGCGGC
fragment 2	34C6	GCCGCCATGACCACCTTTGTATTCGACAACCAGCGGTAC
	28B7	CTATCCCAGGTCTCGTTGTCG
A222Y		
fragment 1	28B6	GTGACGCTCAACCCGGTC
	34D8	GTACCGCTGGTTGTCGAATACATAGGTGGTCATGGCGGC
fragment 2	34D7	GCCGCCATGACCACCTATGTATTCGACAACCAGCGGTAC
	28B7	CTATCCCAGGTCTCGTTGTCG
A222W		
fragment 1	28B6	GTGACGCTCAACCCGGTC
	34D6	GTACCGCTGGTTGTCGAATACCCAGGTGGTCATGGCGGC
fragment 2	34D5	GCCGCCATGACCACCTGGGTATTCGACAACCAGCGGTAC
	28B7	CTATCCCAGGTCTCGTTGTCG

[a] Homology arms for gene cloning are shown in bold. DNA base exchanges introduced for mutations are underlined.

Expression and purification of SmTS1 variants

The transformants containing the mutations were inoculated in LB broth (3 mL, kanamycin). The cultures were grown at 37 °C to form the precultures, the precultures (0.4 mL) were then used to inoculate the expression cultures (400 mL, kanamycin). The expression cultures were incubated at 37 °C until OD_{600} = 0.4–0.6 was reached. The expression cultures were cooled to 18 °C, and IPTG (0.4 mL; 400 mM in H_2O) was added into the expression culture to induce protein expression. Incubation was continued at 18 °C for 16 h.

The cells were collected by centrifugation (3500 rpm × 30 min), and resuspended in binding buffer (7 mL; 50 mM Tris, 300 mM NaCl, 20 mM imidazole, 2 mM β-mercaptoethanol, 1 mM $MgCl_2$, pH = 7.6). The suspension was then lyzed by ultra-sonification (on ice, 6 × 45 s), and the lysates were centrifuged (10000 rpm × 20 min) to remove the cell debris. The supernatant was filtered through a syringe filter (0.45 μm) and loaded onto a Ni^{2+}-NTA affinity chromatography column (Protino™ Ni-NTA, Macherey-Nagel, Düren, Germany). The column was washed with binding buffer (1 mL), washing buffer (2 × 1 mL; 50 mM Tris, 300 mM NaCl, 60 mM imidazole, 2 mM β-mercaptoethanol, 1 mM $MgCl_2$, pH = 7.6), and the protein was obtained by using elution buffer (1 mL; 50 mM Tris, 300 mM NaCl, 300 mM imidazole, 2 mM β-mercaptoethanol, 1 mM $MgCl_2$, pH = 7.6).

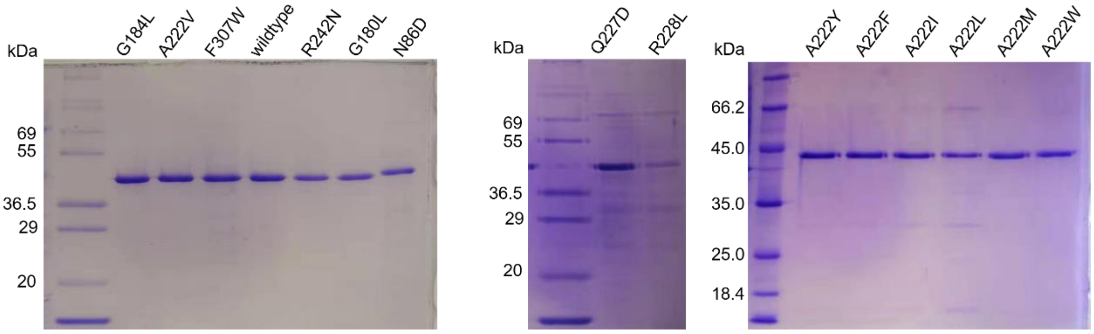

Figure S2. SDS-PAGE analysis of the SmTS1 variants.

Activity of enzyme variants

The protein concentration was determined by the Bradford method,[5] and adjusted to 80 μg mL^{-1} for all variants to test the enzyme activities. The prepared protein solution (200 μL), Tris buffer (200 μl; 50 mM Tris, 1 mM $MgCl_2$, pH = 7.6), incubation buffer (0.5 mL; 50 mM Tris, 10 mM MgCl2, 20% glycerol, pH = 7.6) and GFPP (1 mL; 1 mg mL^{-1} in 25 mM aqueous NH_4CO_3) were mixed and incubated at 28 °C overnight. The products were extracted with hexane (200 μL; containing 0.08 mg L^{-1} octadecane as internal standard) for GC/MS analysis. For every enzyme variant reactions were performed in triplicates.

GC/MS

GC/MS analyses were carried out on a 7890B/5977A series gas chromatography/mass selective detector (Agilent, Santa Clara, CA, USA). The GC was equipped with an HP5-

MS fused silica capillary column (30 m, 0.25 mm i. d., 0.50 μm film; Agilent) and operated using the settings 1) inlet pressure: 77.1 kPa, He at 23.3 mL min^{-1}, 2) injection volume: 1–2 μL, 3) temperature program: 5 min at 50 °C then increasing 5 °C min^{-1} to 320 °C, 4) 60 s valve time, and 5) carrier gas: He at 1.2 mL min^{-1}. The MS was operated with settings 1) source: 230 °C, 2) transfer line: 250 °C, 3) quadrupole: 150 °C and 4) electron energy: 70 eV.

NMR spectroscopy

NMR spectra were recorded at 298 K on a Bruker (Billerica, MA, USA) Avance III HD Cryo (700 MHz) NMR spectrometer. Spectra were measured in C_6D_6 and referenced against solvent signals (^{1}H NMR, residual proton signal: δ = 7.16 ppm; ^{13}C-NMR: δ = 128.06 ppm).[6]

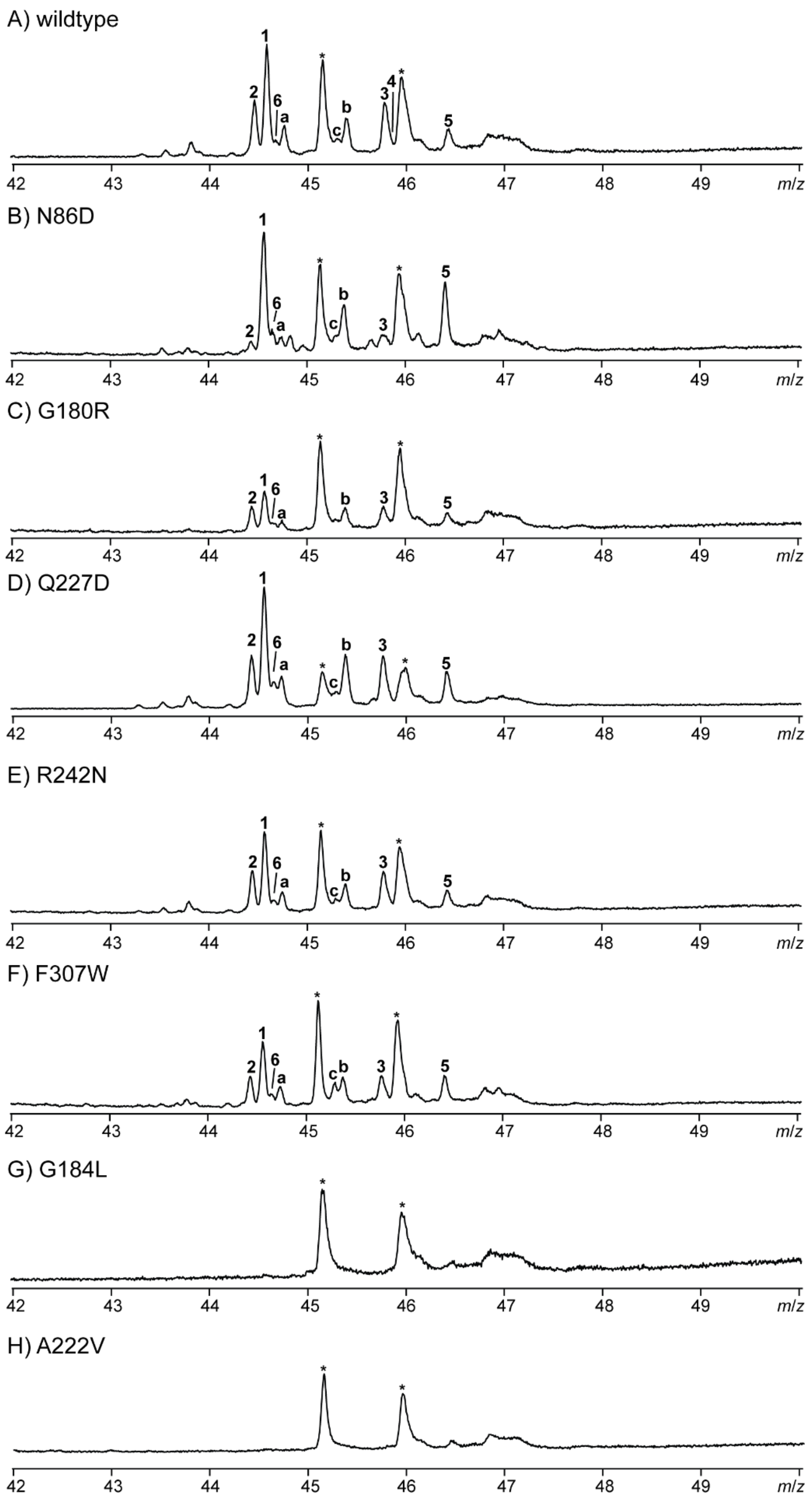

Figure S3. Total ion chromatograms of extracts from incubations of GFPP with A) wildtype SmTS1, and the SmTS1 variants B) N86D, C) G180R, D) Q227D, E) R242N, F) F307W, G) G184L, and H) A222V.

Table S2. Production of compounds **1**–**6** and unknown **a**–**c** by wildtype SmTS1 and its enzyme variants, and relative enzyme activities.

enzyme variant	2	1+6[a]	a	b+c	3+4	5	activity[b]
wildtype	48±3	100±7	25±5	46±7	53±4	30±9	100±19
N86D	15±2	148±9	15±1	68±5	32±3	70±7	104±9
G180R	25±2	41±1	9±1	32±3	35±23	20±5	51±16
Q227D	61±6	163±15	37±4	89±11	79±10	54±12	155±13
R242N	54±6	105±9	24±3	51±7	60±14	25±4	94±17
F307W	23±2	54±5	17±1	39±3	26±3	23±3	53±6
A222V	–	–	–	–	–	–	–
G184L	–	–	–	–	–	–	–

[a] Production of compounds **1** – **6** by peak integration of total ion chromatograms from triplicates. Production of **1** and co-eluting **6** by wildtype SmTS1 is set to 100%. [b] Enzyme activities were calculated from total production of all compounds by peak integrations from triplicates. Activity of wildtype SmTS1 is set to 100%.

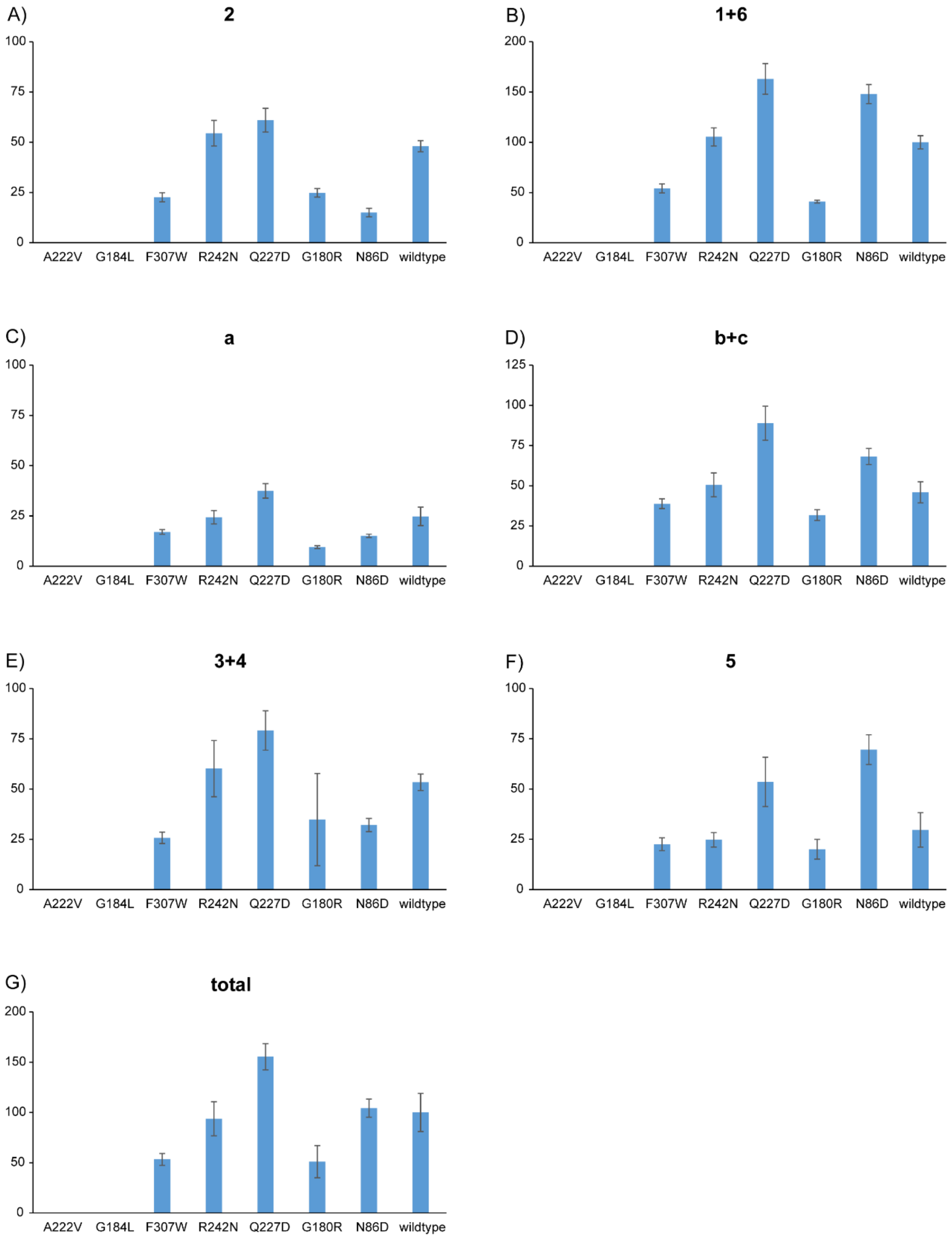

Figure S4. Production of compounds **1**–**6** and **a**–**c** by peak integration of total ion chromatograms (mean ± standard deviation from triplicates). Production of **1** with co-eluting **6** by wildtype SmTS1 is set to 100%. The production of A) **2**, B) **1** + **6**, C) unknown **a**, D) unknown **b** + **c**, E) **3** + **4**, F) **5** and G) total activities of the wildtype and each variant. For a comparison of enzyme activities, the peak integrals of all produced compounds were summarised. Activity of wildtype SmTS1 is set to 100%.

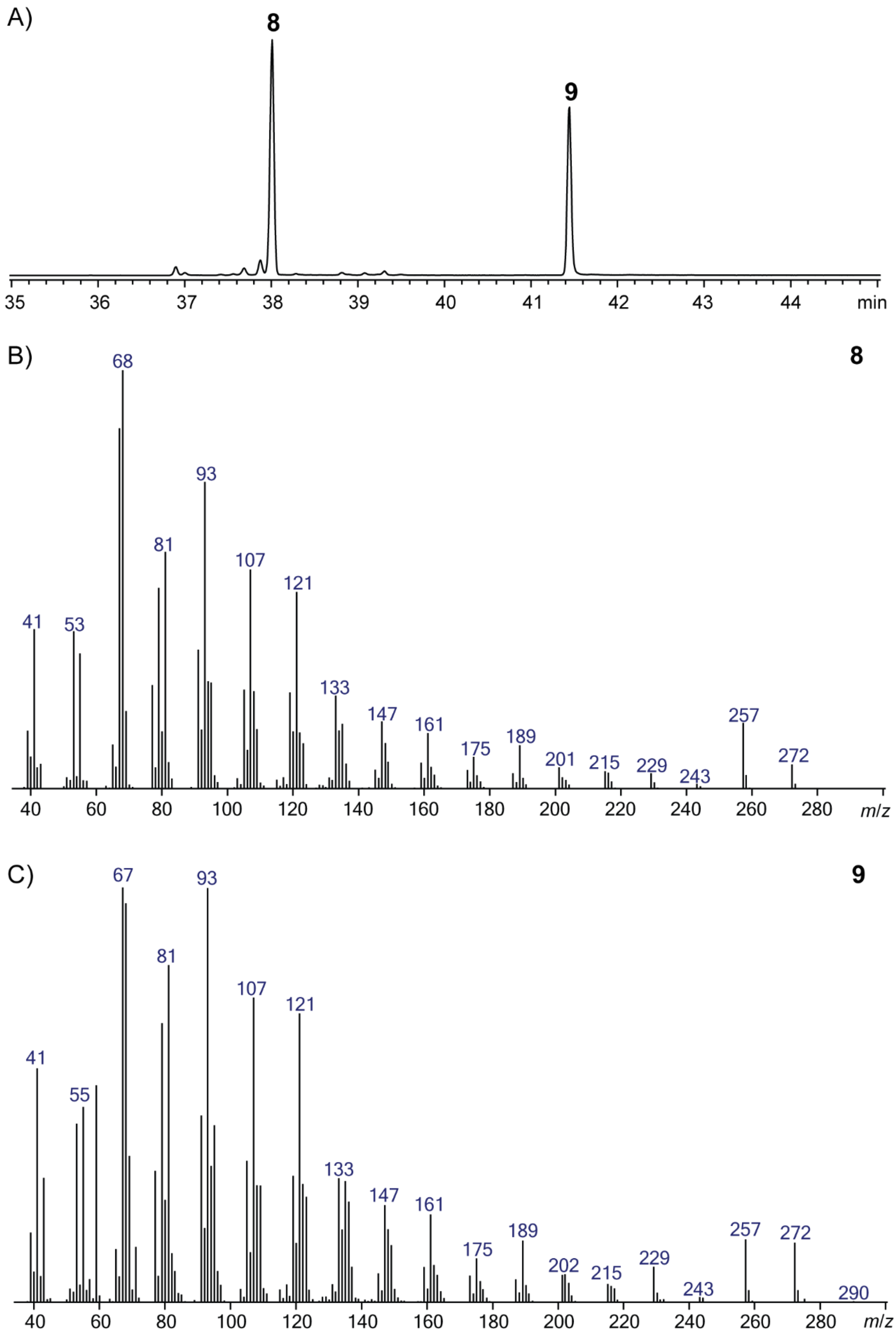

Figure S5. The A222V enzyme variant. A) Total ion chromatogram of an extract from an incubation with GGPP, and mass spectra of B) **8** and C) **9**.

Table S3. Production of sesterterpens and and diterpenes by wildtype SmTS1 and its enzyme variants related to the position A222, and relative enzyme activities.

enzyme variant	sesterterpenes[a]	8	9	diterpenes[b]
Wildtype	100±8%	0	0	0
A222V	0	98±11%	31±2%	129±8%
A222M	39±9%	82±3%	80±2%	162±3%
A222I	0	110±2%	47±2%	157±1%
A222L	13±4%	89±3%	54±8%	142±11%
A222F	0	21±2%	15±8%	37±8%

[a] Production of sesterterpenes by wildtype SmTS1 is set to 100%. [b] Enzyme activities were calculated from total production of **8** and **9** by peak integrations from triplicates.

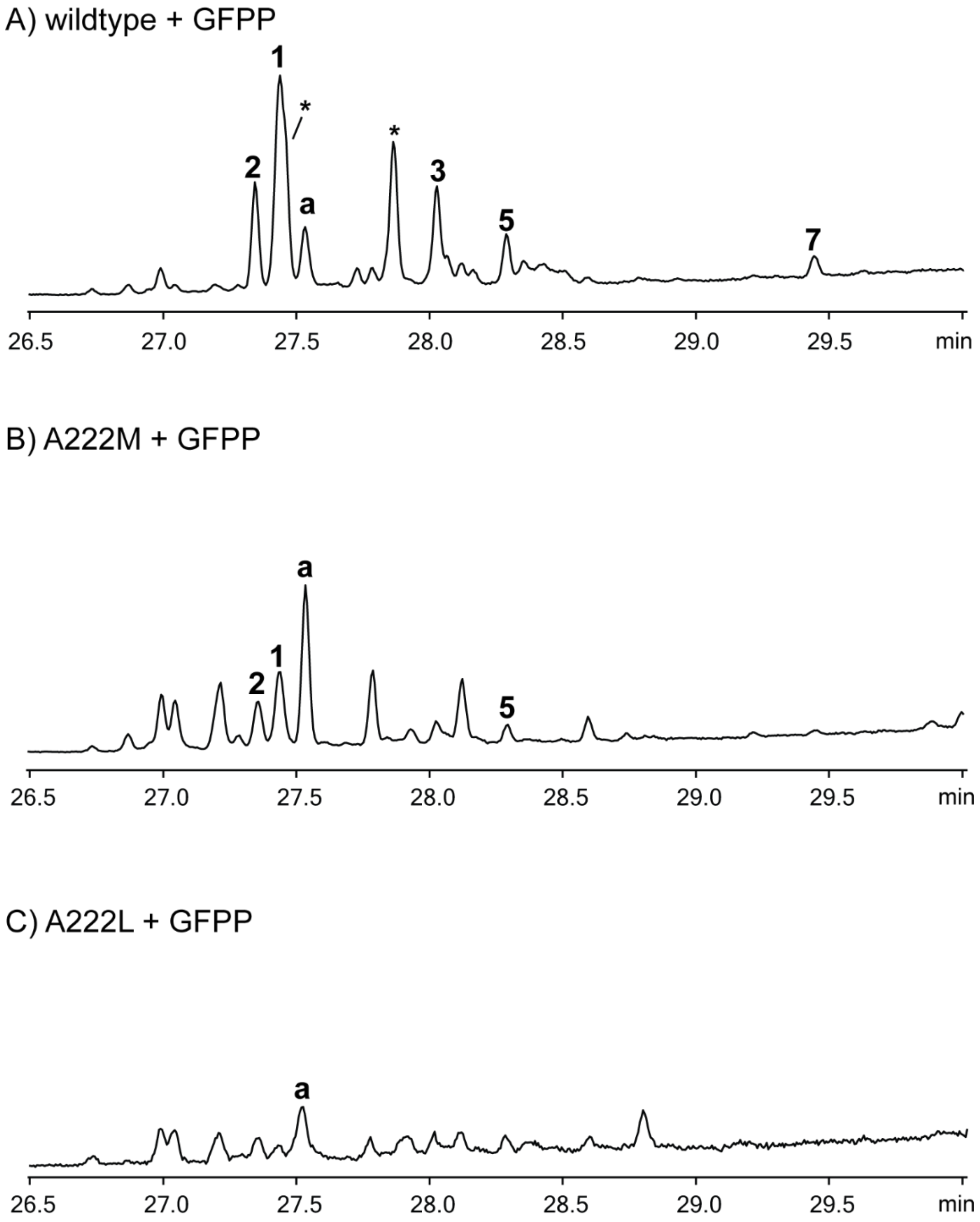

Figure S6. Total ion chromatograms of extracts from incubations of GFPP with A) wildtype SmTS1, and the SmTS1 variants B) A222M, and C) A222L.

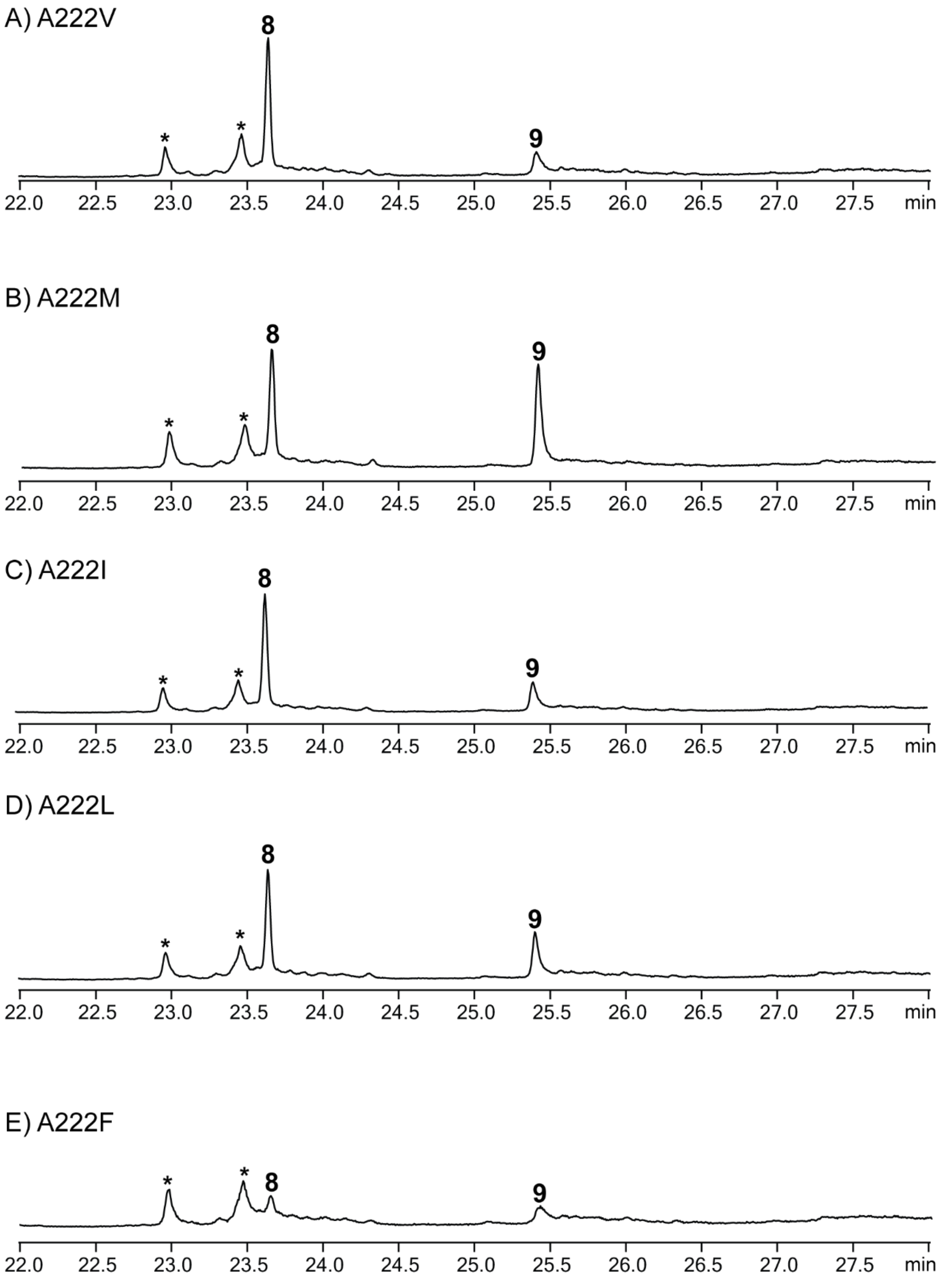

Figure S7. Total ion chromatograms of extracts from incubations of GGPP with the SmTS1 variants A) A222V, B) A222M, C) A222I, D) A222L, and E) A222F. Asterisks indicate degradation products from GGPP also observed without enzyme.

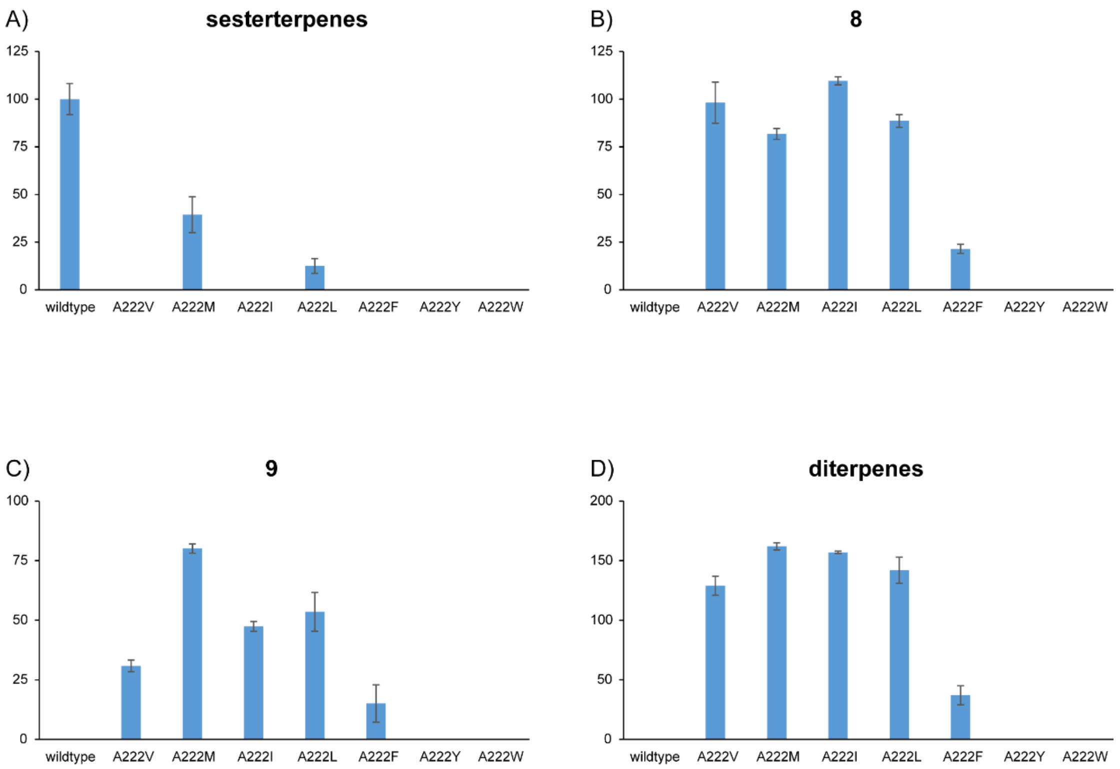

Figure S8. Production of sesterterpenes and diterpenes from SmTS1 and its variants in the position of A222. Production of A) sesterterpenes, B) compound **8**, C) compound **9** and D) diterpenes (sum of **8** and **9**) by the wildtype and its variants. The data were obtained by peak integration of total ion chromatograms (mean ± standard deviation from triplicates). Production of sesterterpenes by wildtype is set to 100%.

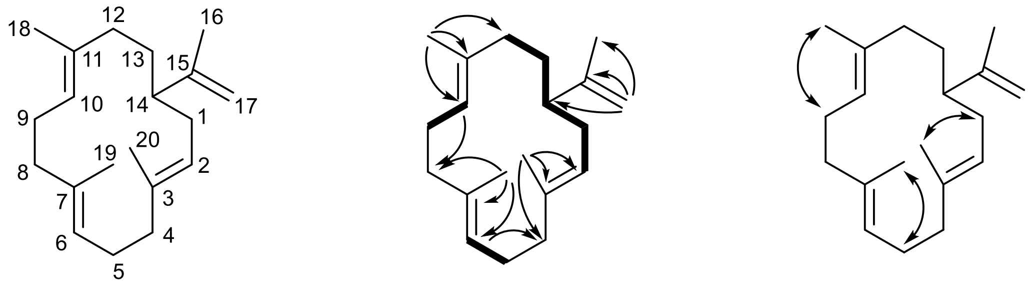

8

Figure S9. Structure elucidation of **8**. Bold: $^{1}H,^{1}H$ COSY, single headed arrows: key HMBC, and double headed arrows: key NOESY correlations. Carbon numbering follows GGPP numbering to indicate the origin of each carbon.

Table S4. NMR data of cembrene A (**8**) in C_6D_6 recorded at 298 K.

C[a]	^{13}C[b]	^{1}H[b]
1	33.02	2.16 (m, 1H); 2.02 (m, 1H)
2	124.57	5.32 (ddq, *J* = 8.1, 6.7, 1.3 Hz, 1H)
3	134.91	–
4	39.38	2.15 (m ,1H)
		2.11 (m, 1H)
5	25.35	2.26 (m, 1H)
		2.13 (m, 1H)
6	126.45	5.08 (dddd, *J* = 7.0, 5.5, 2.6, 1.3 Hz, 1H)
7	133.48	–
8	39.85	2.10 (m, 2H)
9	24.22	2.15 (m, 2H)
10	122.33	5.25 (ddq, *J* = 6.5, 4.6, 1.5 Hz, 1H)
11	133.86	–
12	34.37	2.04 (m, 1H)
		1.94 (m, 1H)
13	28.59	1.80 (ddt, *J* = 13.9, 10.0, 3.9 Hz, 1H)
		1.41 (dddd, *J* = 13.8, 10.1, 6.7, 3.8 Hz, 1H)
14	46.55	2.22 (m, 1H)
15	149.25	–
16	19.37	1.63 (t, *J* = 1.2 Hz, 3H)
17	110.80	4.83 (m, 2H)
18	18.33	1.60 (s, 3H)
19	15.40	1.54 (s, 3H)
20	15.64	1.55 (s, 3H)

[a] Carbon numbering as shown in Figure S9. [b] Chemical shifts δ in ppm, multiplicity: s = singlet, d = doublet, t = triplet, q = quartet, m = multiplet.

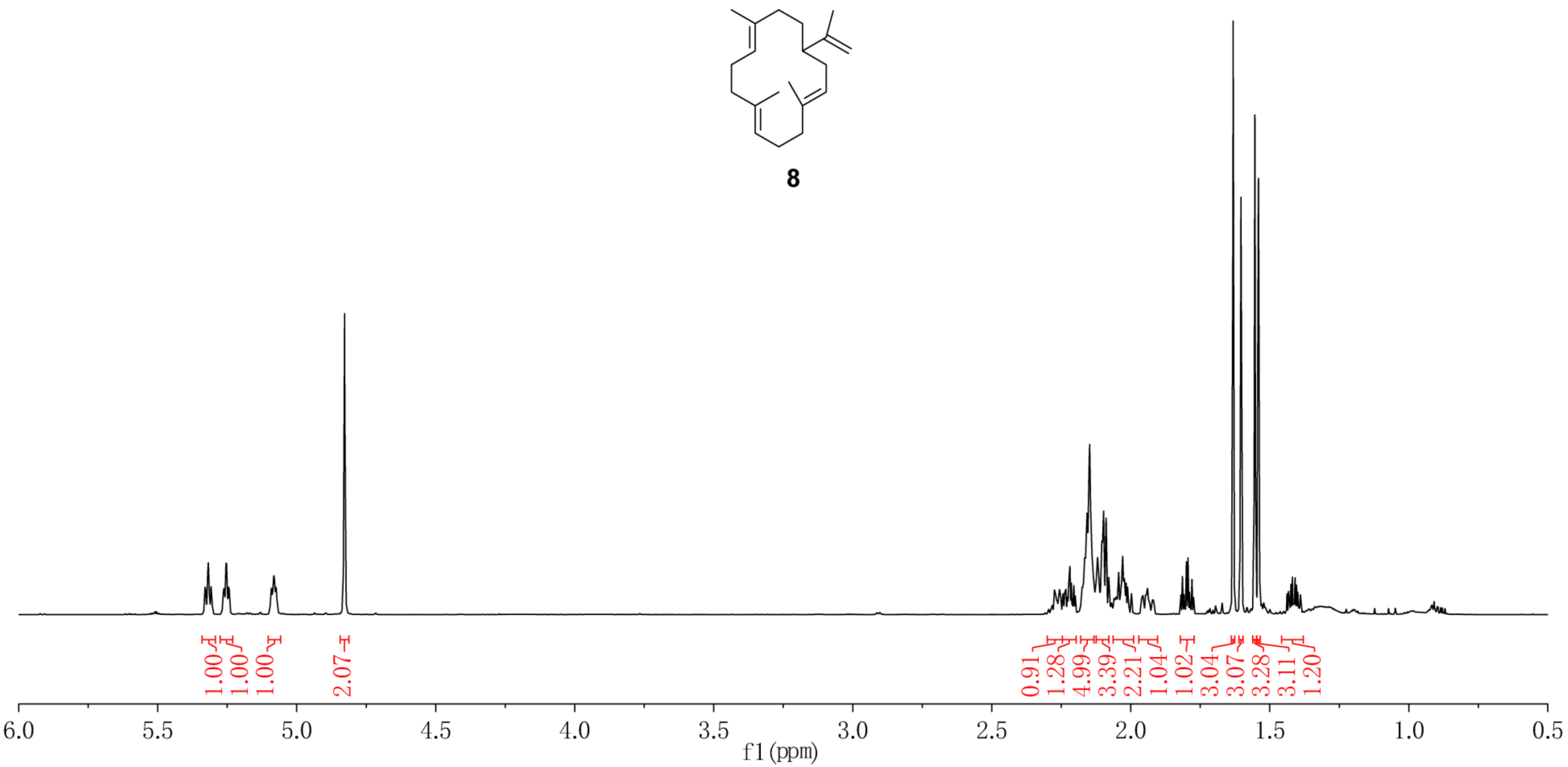

Figure S10. ^{1}H NMR spectrum of **8** (700 MHz, C_6D_6).

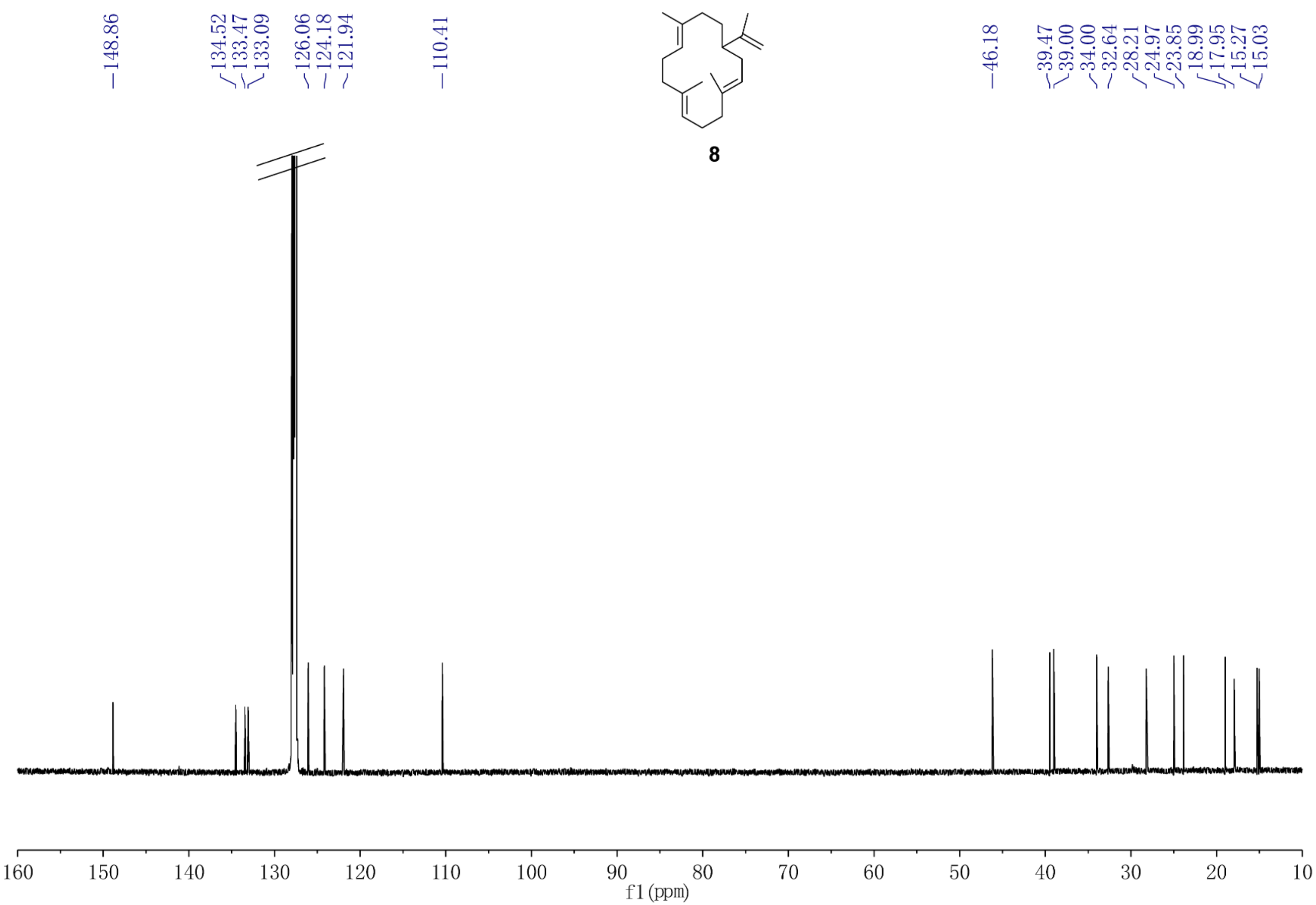

Figure S11. ^{13}C NMR spectrum of **8** (176 MHz, C_6D_6).

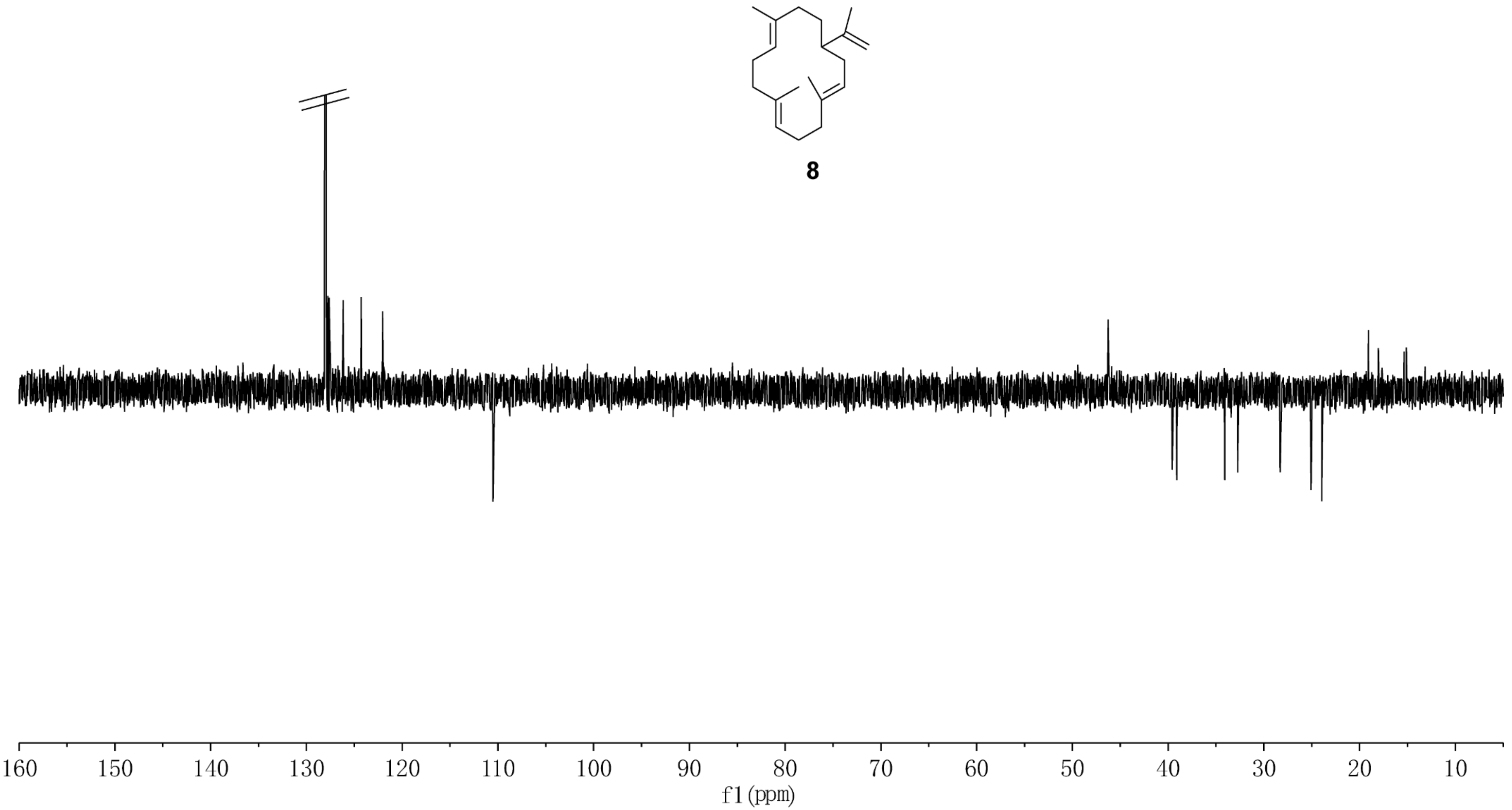

Figure S12. ^{13}C DEPT spectrum of **8** (176 MHz, C_6D_6).

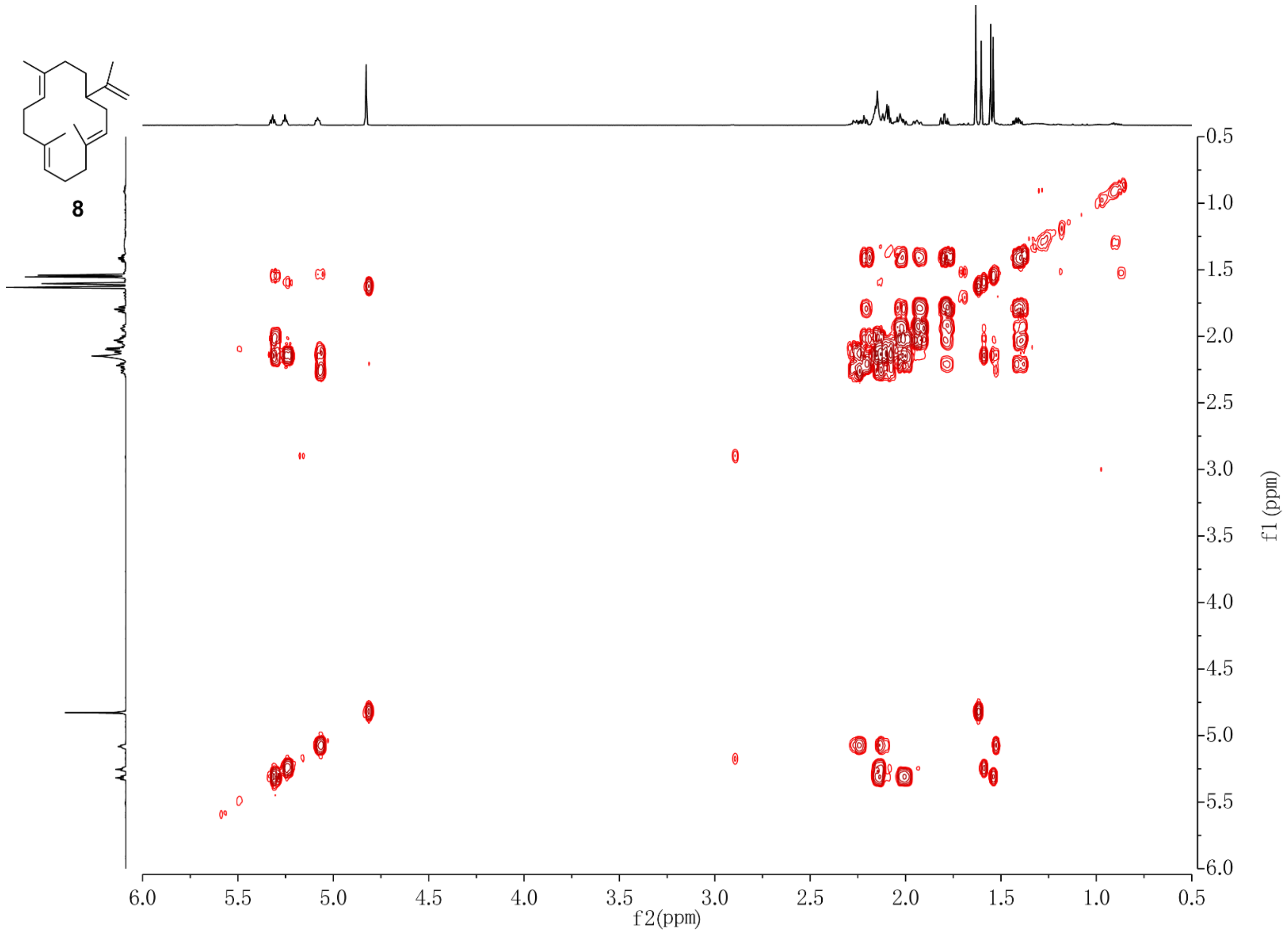

Figure S13. $^{1}H,^{1}H$ COSY spectrum of **8** (700 MHz, C_6D_6).

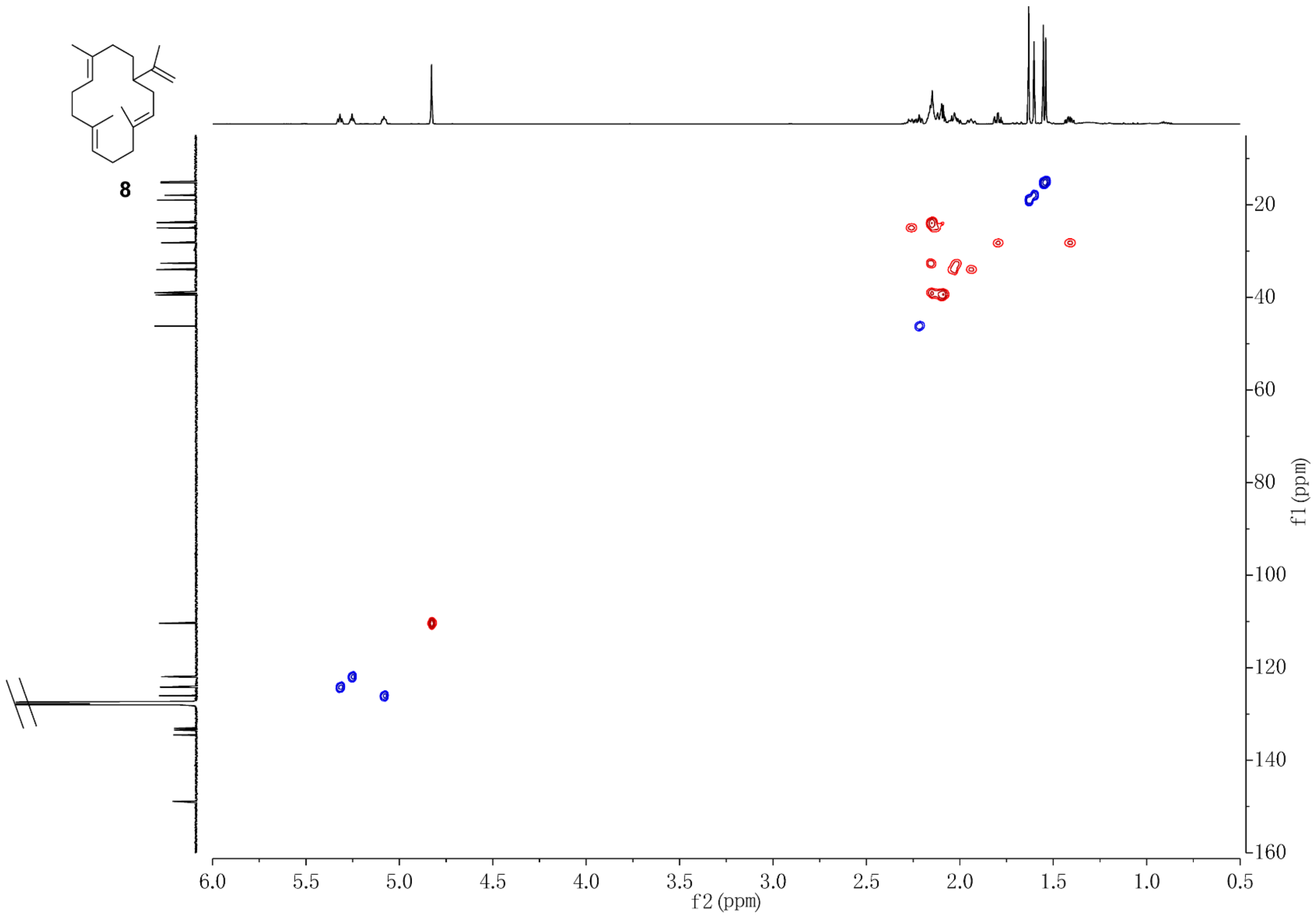

Figure S14. HSQC spectrum of **8** (C_6D_6).

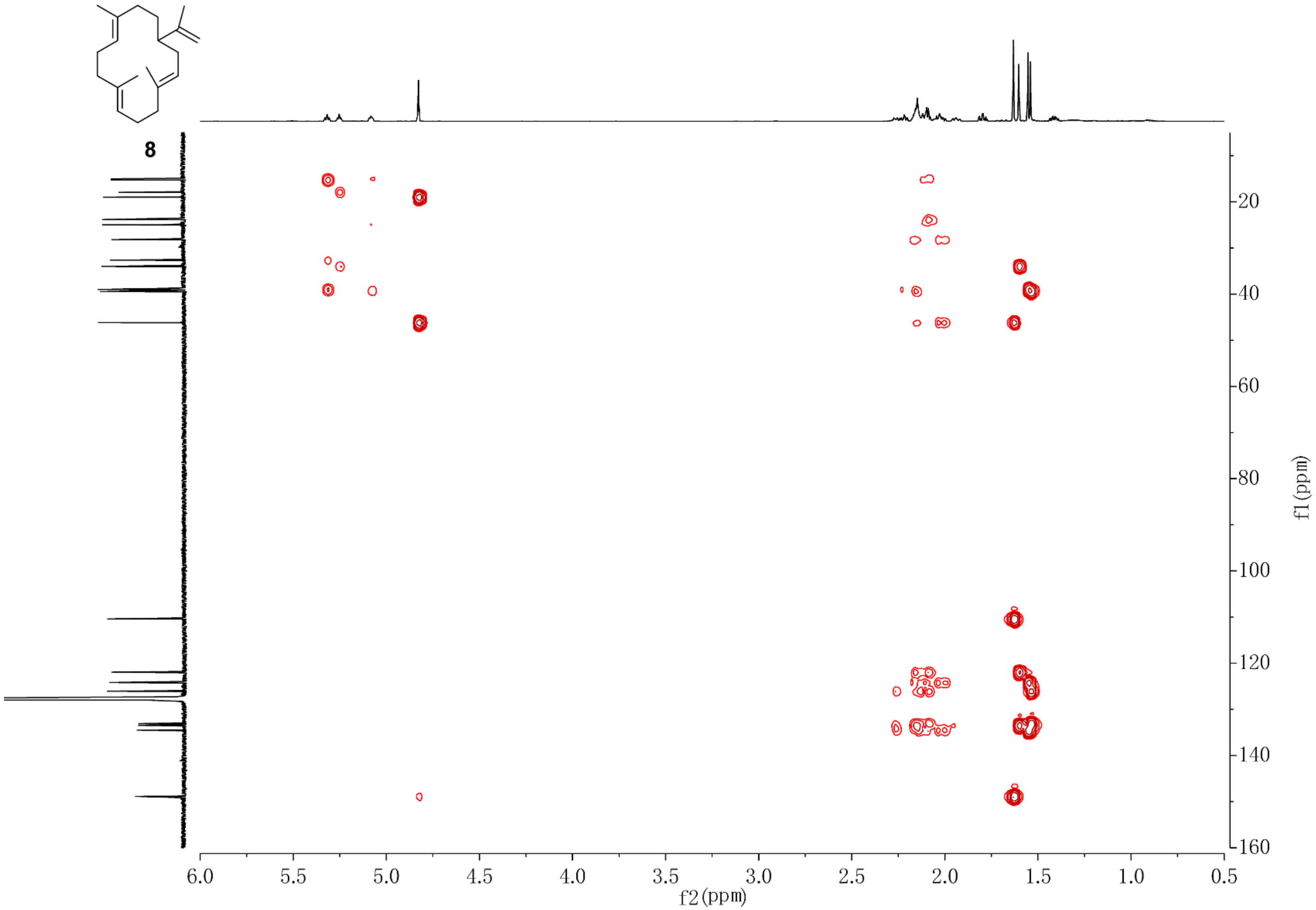

Figure S15. HMBC spectrum of **8** (C_6D_6).

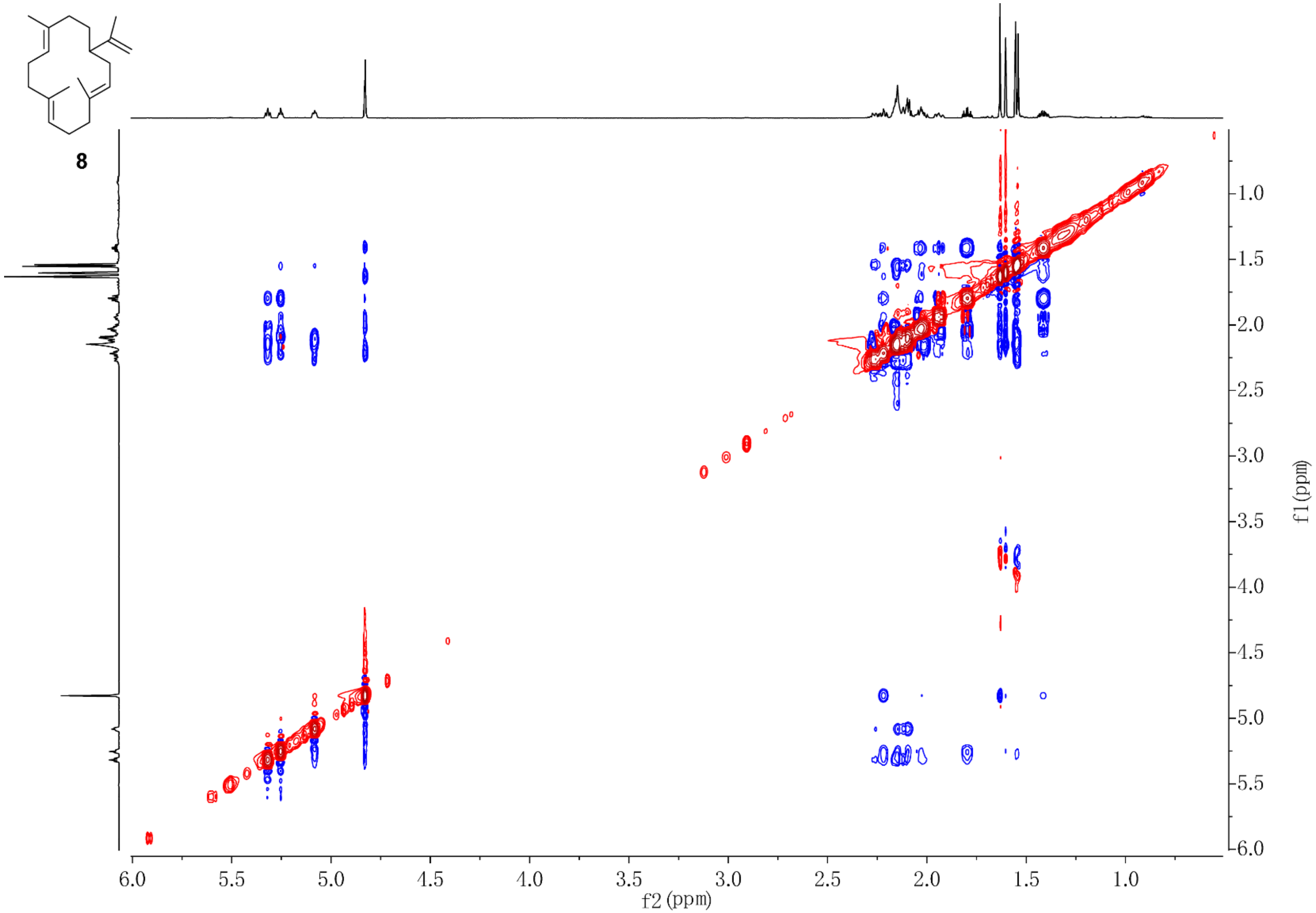

Figure S16. NOESY spectrum of **8** (C_6D_6).

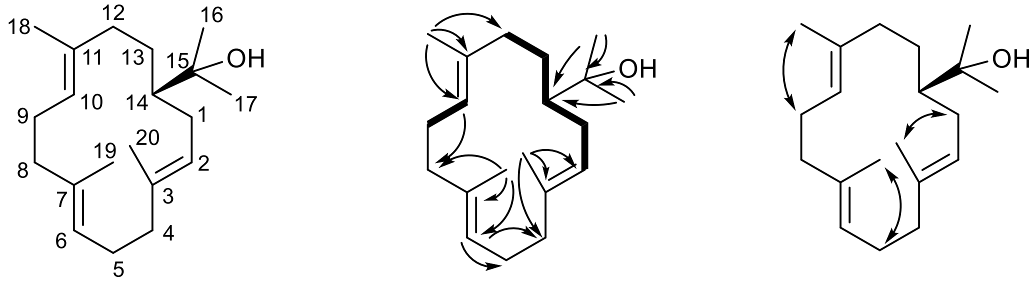

9

Figure S17. Structure elucidation of **9**. Bold: $^{1}H,^{1}H$-COSY, single headed arrows: key HMBC, and double headed arrows: key NOESY correlations. Carbon numbering follows GGPP numbering to indicate the origin of each carbon.

Table S5. NMR data of (*R*)-nepthenol (**9**) in C_6D_6 recorded at 298 K.

C[a]	^{13}C[b]	^{1}H[b]
1	29.04	2.20 (m, 1H)
		1.91 (dt, *J* = 15.1, 7.6 Hz, 1H)
2	126.93	5.26 (tq, *J* = 7.2, 1.3 Hz, 1H)
3	133.04	–
4	39.31	2.13 (m, 2H)
5	25.16	2.19 (m, 2H)
6	126.29	5.07 (t, J = 6.7 Hz, 1H)
7	133.02	–
8	39.88	2.10 (m, 1H)
		2.06 (m, 1H)
9	24.49	2.14 (m, 2H)
10	125.33	5.16 (t, *J* = 6.8 Hz, 1H)
11	134.28	–
12	38.21	2.16 (m, 1H)
		2.09 (m, 1H)
13	28.77	1.66 (m, 1H)
		1.24 (m, 1H)
14	48.8	1.34 (m, 1H)
15	73.21	–
16	27.85	1.07 (s, 3H)
17	27.88	1.06 (s, 3H)
18	15.79	1.59 (s, 3H)
19	15.44	1.54 (d, *J* = 1.3 Hz, 3H)
20	15.69	1.57 (s, 3H)

[a] Carbon numbering as shown in Figure S17. [b] Chemical shifts δ in ppm, multiplicity: s = singlet, d = doublet, t = triplet, q = quartet, m = multiplet.

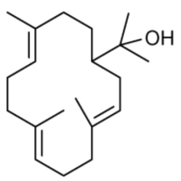

9

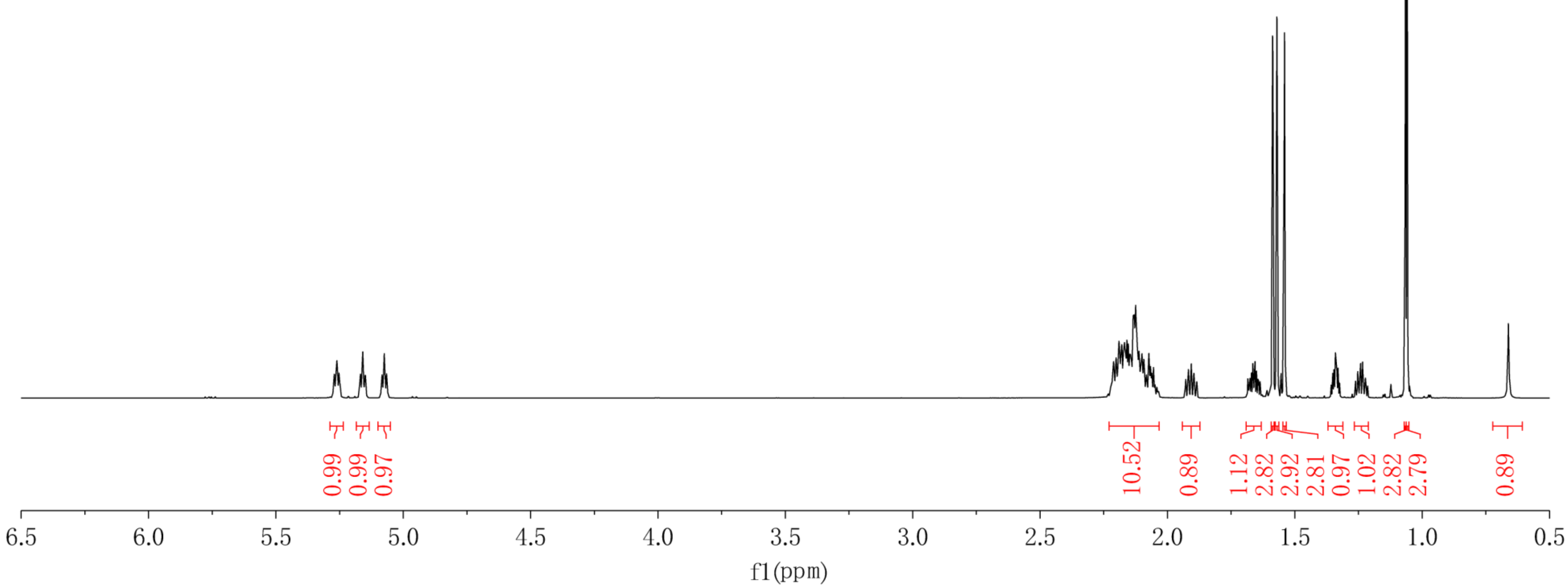

Figure S18. ^{1}H NMR spectrum of **9** (700 MHz, C_6D_6).

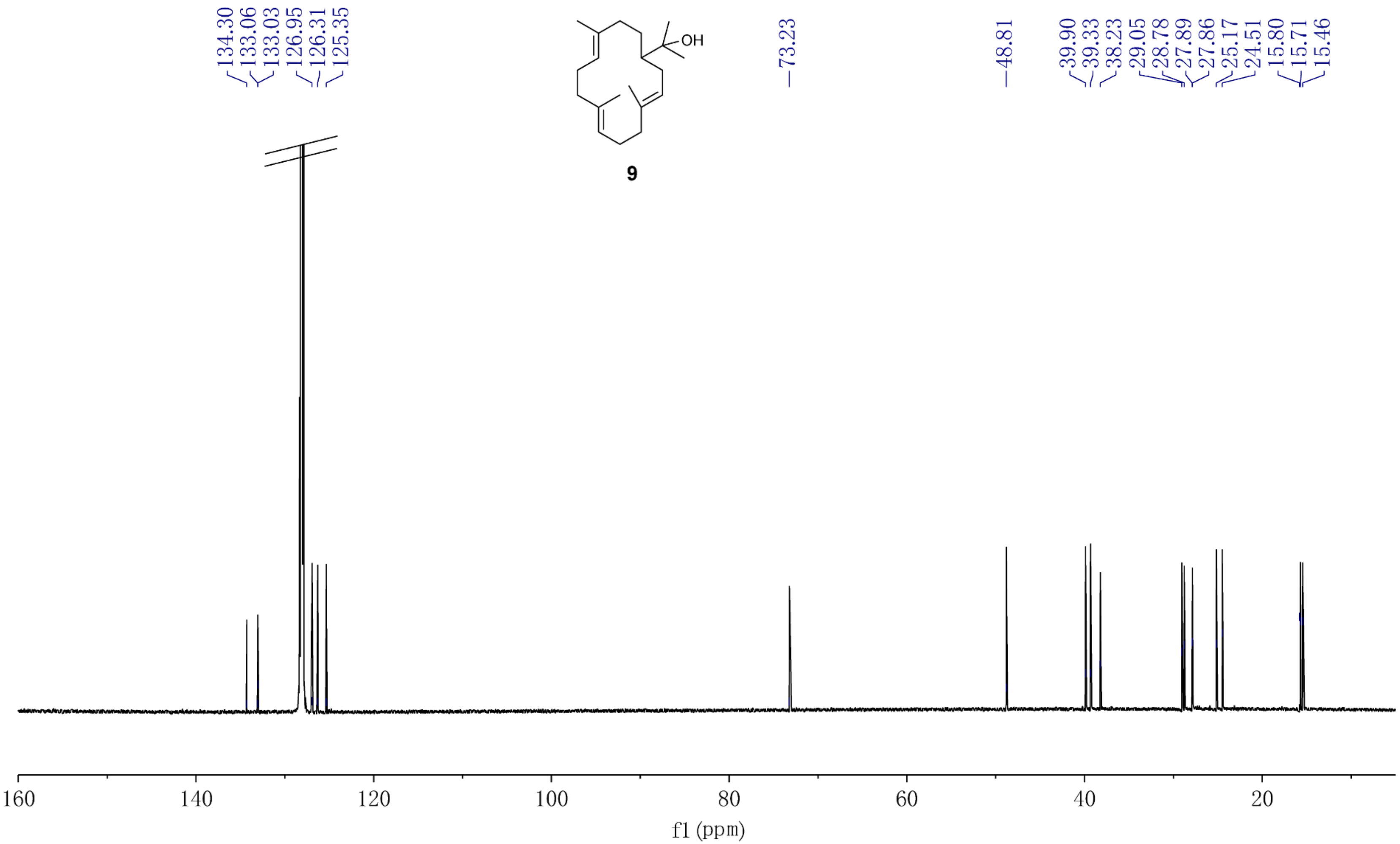

Figure S19. ^{13}C NMR spectrum of **9** (176 MHz, C_6D_6).

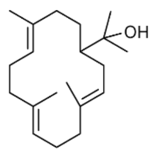

9

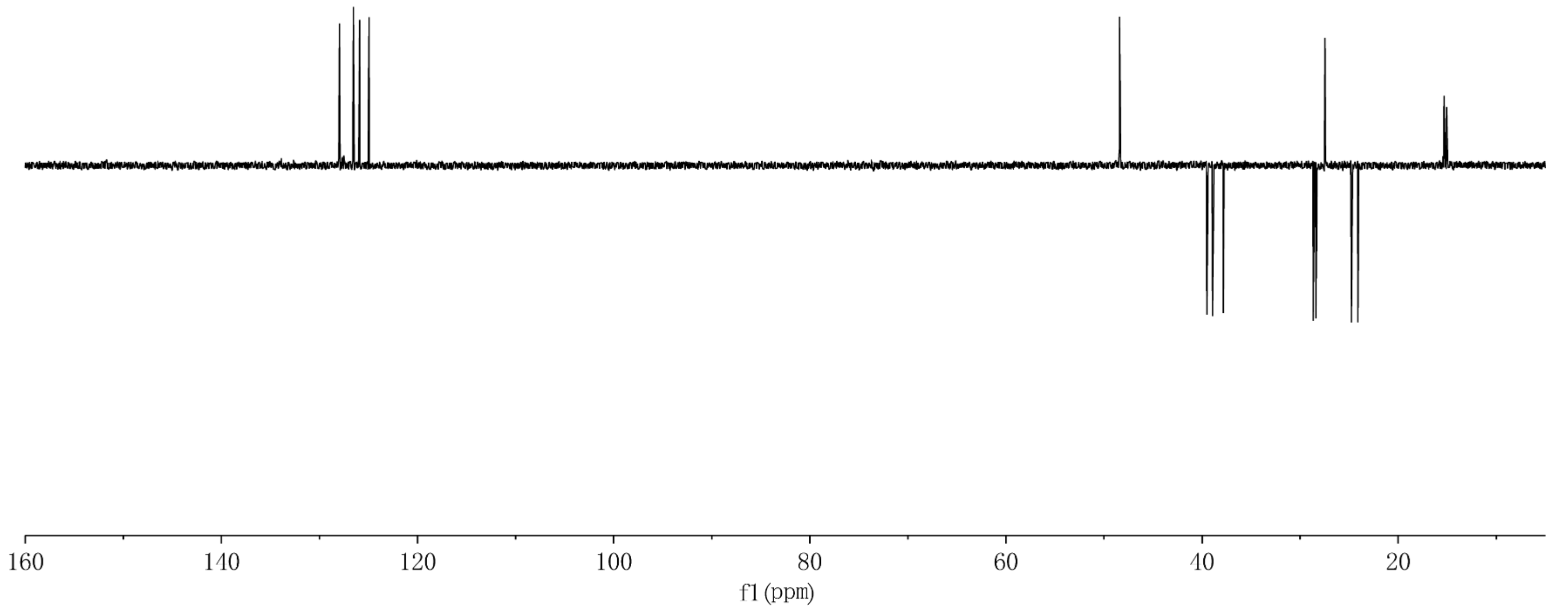

Figure S20. ^{13}C DEPT spectrum of **9** (176 MHz, C_6D_6).

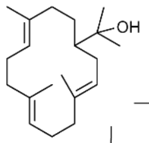

9

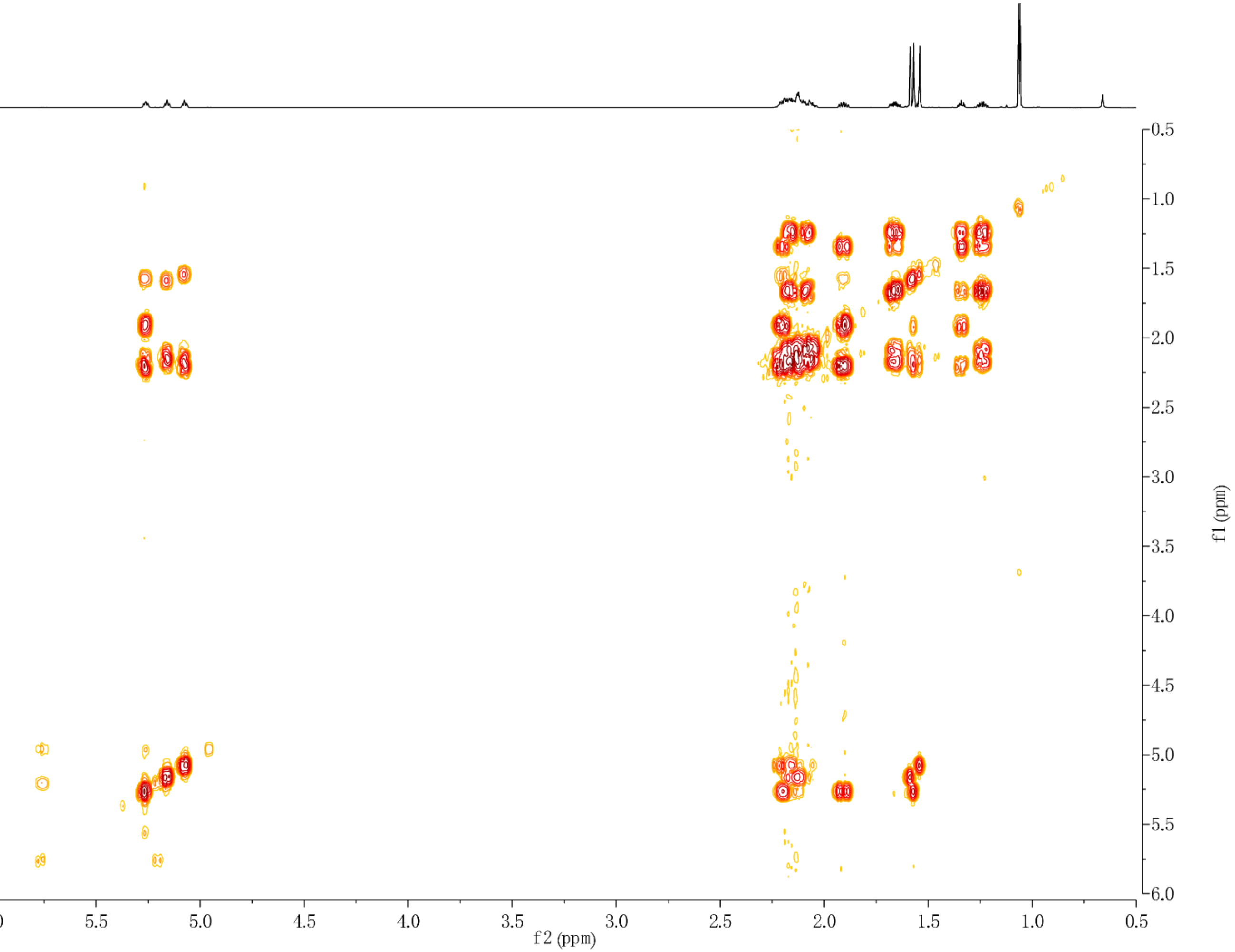

Figure S21. $^{1}H,^{1}H$ COSY spectrum of **9** (700 MHz, C_6D_6).

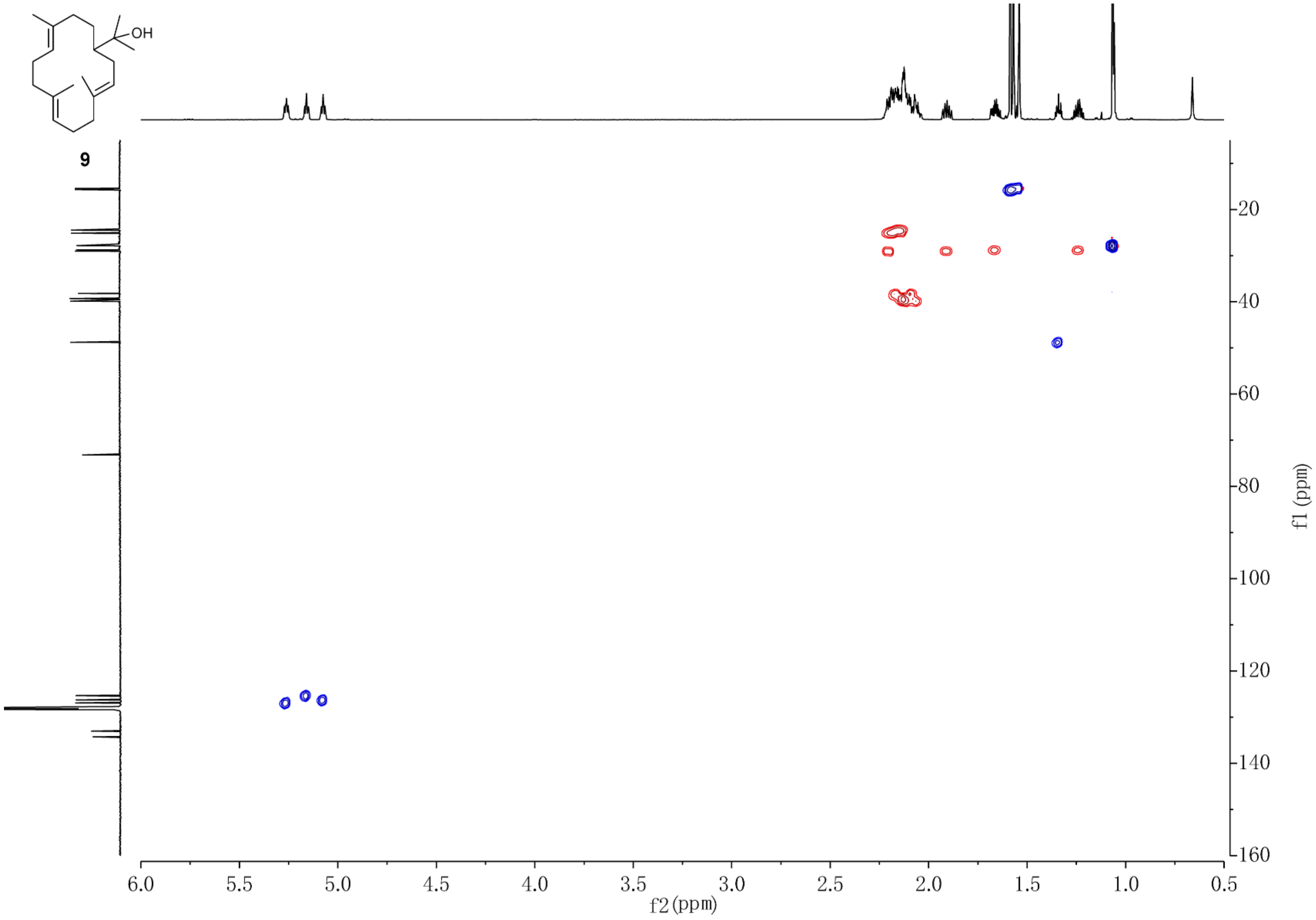

Figure S22. HSQC spectrum of **9** (C_6D_6).

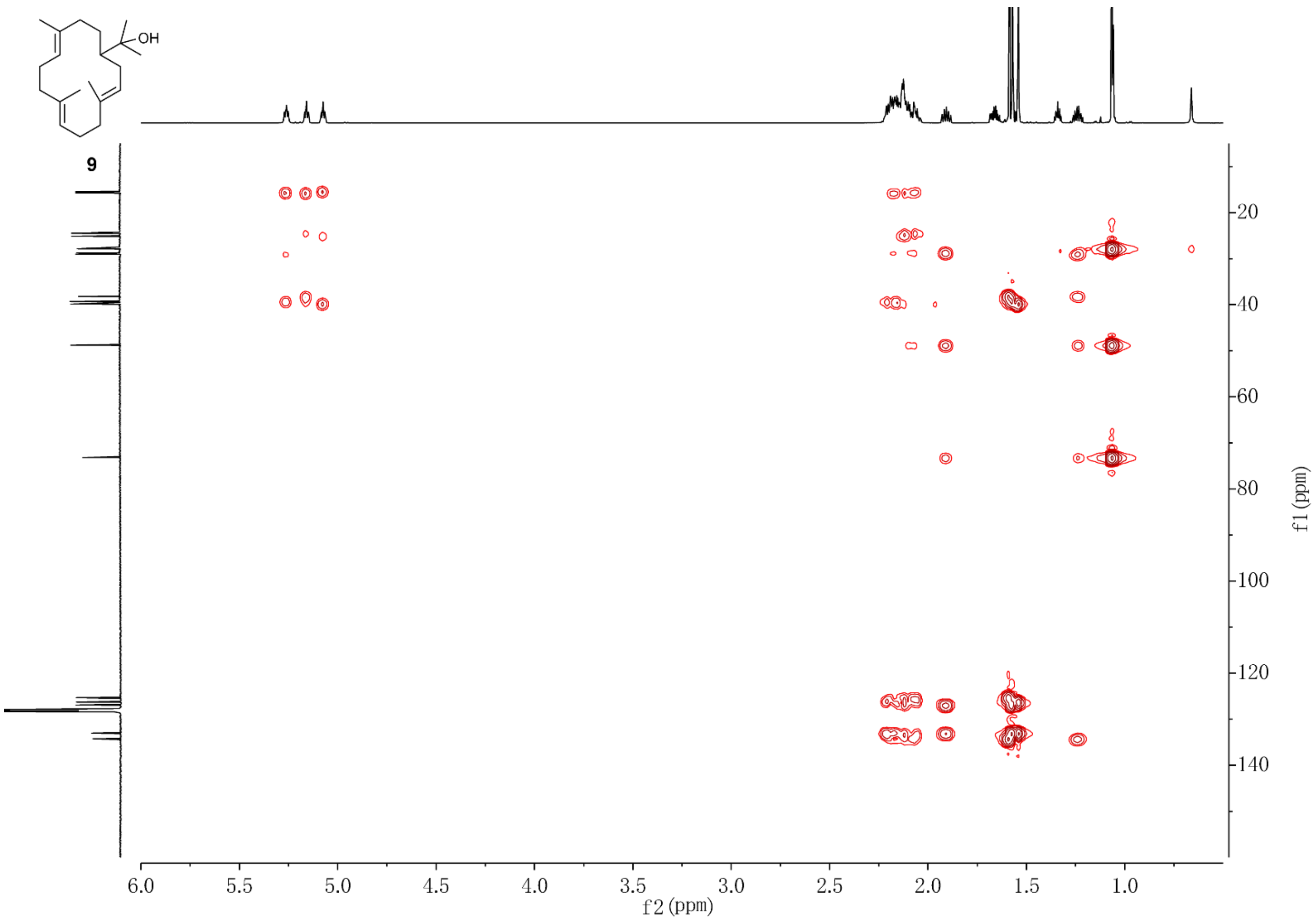

Figure S23. HMBC spectrum of **9** (C_6D_6).

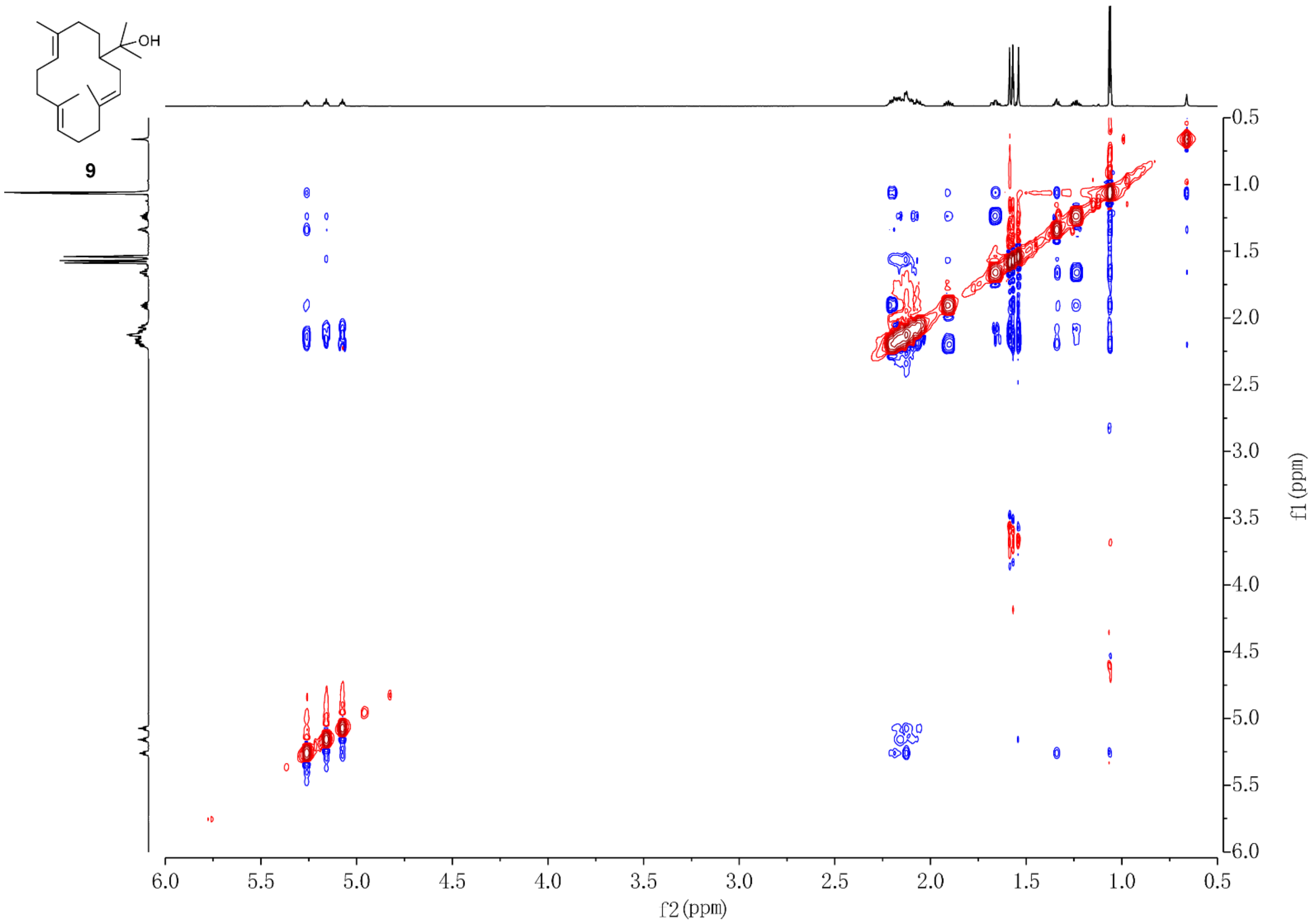

Figure S24. NOESY spectrum of **9** (C_6D_6).

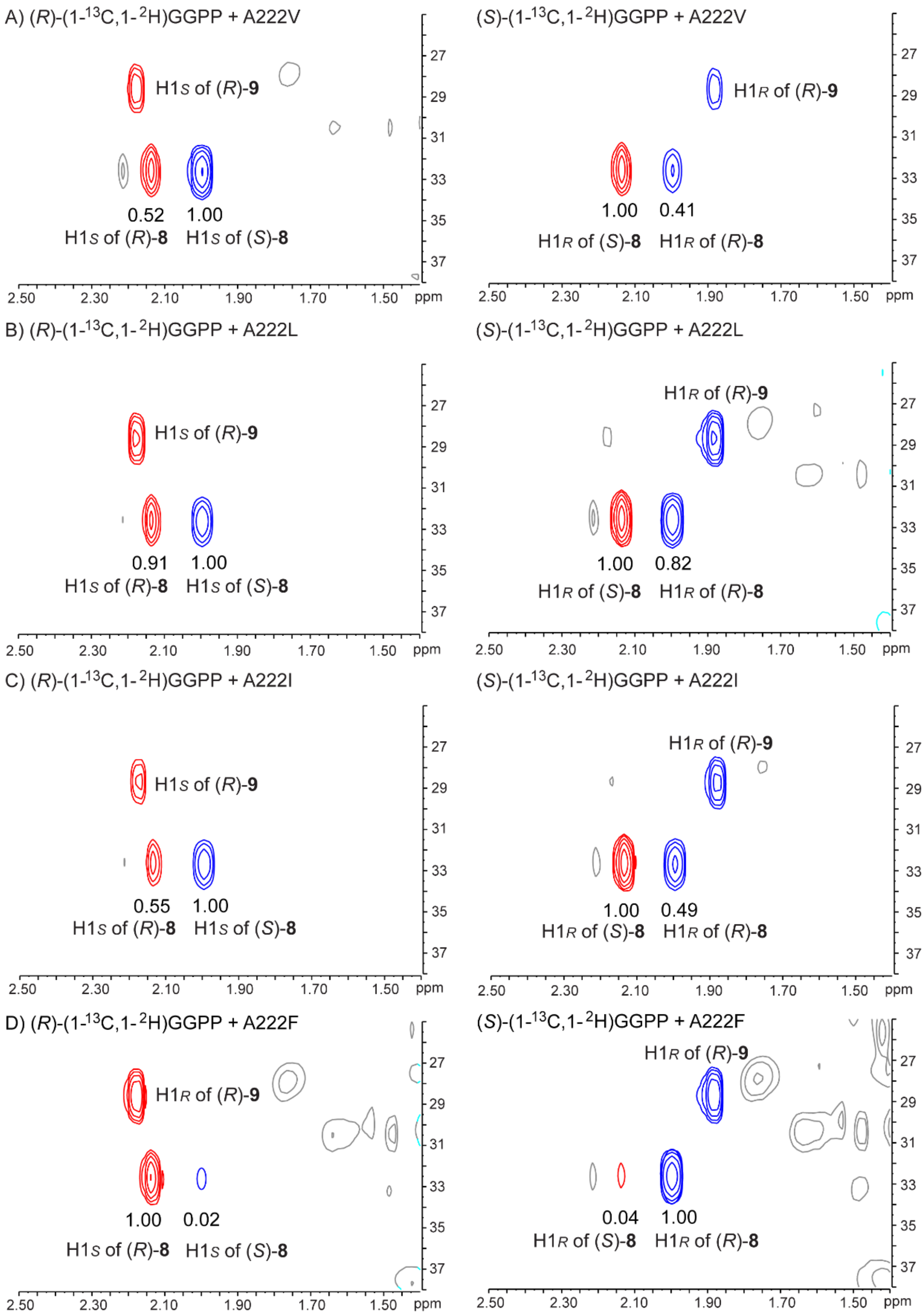

Figure S25. Determination of the absolute configuration of compounds **8** and **9** from SmTS1 variants. Partial HSQC spectra of labelled **8** and **9** obtained from (*R*)-(1-^{13}C,1-^{2}H)GGPP (left) and (*S*)-(1-^{13}C,1-^{2}H)GGPP (right) with variant A) A222V, B) A222L, C) A222I, and D) A222F.

References

1. Higuchi, R.; Krummel, B.; Saiki, R. *Nucleic Acids Res.,* **1988,** *16,* 7351–7367.
2. Hou, A.; Dickschat, J. S. *Angew. Chem. Int. Ed.,* **2020,** *59,* 19961–19965.
3. Giets, R. D.; Schiestl, R. H. *Nat. Protoc.,* **2007,** *2,* 31–34.
4. Dickschat, J. S.; Pahirulzaman, K. A. K.; Rabe, P.; Klapschinski, T. A. *ChemBioChem,* **2014,** *15,* 810–814.
5. Bradford, M. M. *Anal. Biochem.,* **1976,** *72,* 248–254.
6. Fulmer, G. R.; Miller, A. J. M.; Sherden, N. H.; Gottlieb, H. E.; Nudelman, A.; Stoltz, B. M.; Bercaw, J. E.; Goldberg, K. I. *Organometallics,* **2010,** *29,* 2176–2179.
